# Tart cherry supplementation causes differential regulation of skeletal muscle proteome after eccentric exercise

**DOI:** 10.3389/fnut.2026.1801399

**Published:** 2026-05-26

**Authors:** Vlad Sabou, Mary O'Leary, Sarah R. Jackman, Lauren Struszczak, Esra Bozbas, Brad Metcalf, Jonathan C. Y. Tang, Mark Philo, Paul A. Kroon, Joanna L. Bowtell

**Affiliations:** 1 Department of Sport and Health Sciences, University of Exeter, St Luke's Campus, Exeter, United Kingdom; 2 Bioanalytical Facility, Norwich Medical School, University of East Anglia, Norwich, United Kingdom; 3 Department of Clinical Biochemistry, Norfolk and Norwich University Hospital NHS Foundation Trust, Colney Lane, Norwich, United Kingdom; 4 Food, Microbiome and Health, Quadram Institute, Norwich Research Park, Norwich, United Kingdom

**Keywords:** exercise recovery, muscle damage, muscle proteome, polyphenols, tart cherry

## Abstract

Polyphenol-rich tart cherry (TC) supplementation has previously been shown to enhance recovery from exercise-induced muscle damage (EIMD). However, no studies have investigated the effects of different doses of TC supplementation on such recovery. The molecular mechanism(s) of action through which TC elicits these effects is unknown. Here we show that 7 days TC supplementation prior to EIMD increases TC-derived plasma phenolic acid concentrations and profoundly alters the balance of structural and contractile muscle proteins, and macrophage cell infiltration within the exercised muscle. We demonstrate that plasma hippuric acid concentrations are positively correlated with muscle function in young active males. These molecular changes occurred in the absence of TC-accelerated recovery of muscle function. These findings suggest that TC warrants exploration as an intervention that may enhance musculoskeletal adaptations to training stimuli.

**Clinical Trial Registration:**

https://clinicaltrials.gov/study/NCT04725149, identifier: NCT04725149.

## Introduction

Exercise involving strenuous eccentric muscle contractions and explosive movements leads to muscle damage (1–3). Exercise-induced muscle damage (EIMD) can last for multiple days following an exercise bout and is characterized by a substantial decrease in muscle strength and exercise capacity (4, 5), as well as soreness and swelling of the involved muscles (4, 6). Although the processes involved in muscle damage are not fully characterized, there are two phases, which involve mechanical, metabolic and immune pathways (7).

High mechanical forces as well as reactive oxygen and nitrogen species (RONS) are generated during eccentric exercise which lead to the initial exercise-induced damage (8). The “popping sarcomere” has been the prevailing theory whereby passive elongation of the sarcomere is non-uniform, and weaker sarcomeres are excessively stretched and rupture or “pop” (8), leading to calcium influx into the sarcoplasm, activating calpains and causing muscle damage (9). However, the intramuscular extracellular matrix (ECM) which provides mechanical support to muscle tissue and nerves (10) and plays a key role in the transmission of contractile force is also disrupted by damaging eccentric exercise (8). Therefore, an integrated “shear deformation theory” has recently been proposed in which titin and the extracellular matrix play a pivotal role (11). A-band myosin is attached to Z-disk actin by titin. When the sarcomere length exceeds the physiological range, stiffening of titin and the ECM occurs to protect sarcolemmal integrity (12). This stiffening increases lateral transmission of forces through the ECM, causing shear deformation and damage to both the ECM and sarcolemma. In common with the “popping sarcomere” theory, the resulting increase in sarcolemmal permeability allows an influx of Ca^2+^ into the sarcoplasm thus activating calpains and triggering ultrastructural damage, compromising excitation-contraction coupling (ECC), and impairing function. An early proteomics study supports the notion that the ECM is involved in the response to skeletal muscle damage. Two-dimensional gel electrophoresis identified the involvement of Z band proteins desmin, actin (increased expression), and LIM domain-binding protein 3 (reduced expression) in the muscle damage response; and altered expression of calsequestrin-1 consistent with disrupted ECC (13). Whilst these data support the involvement of the ECM in the skeletal muscle damage response, modern proteomics techniques can now facilitate a much more comprehensive analysis.

Following the initial phase of mechanical and metabolic damage, immune cells infiltrate skeletal muscle; initially neutrophils that peak at 24 h post-damage. This results in subsequent mobilization of other immune cells, primarily macrophages. Immune cells contribute to the evolving skeletal muscle injury via release of RONS, proteolytic enzymes and inflammatory cytokines. Phagocytic immune cells clear this accumulating debris. Alongside mechanical damage, exercise performance is negatively affected by increased oxidative stress; oxidative stress is thought to decrease muscle force production due to impaired calcium handling and myofibrillar calcium sensitivity (14, 15). Given the central role of oxidative stress and inflammation in the muscle damage process, nutritional strategies that induce antioxidant and anti-inflammatory effects represent plausible solutions for reducing exercise-induced muscle damage and its associated recovery time.

Montmorency tart cherries are a rich source of polyphenols shown to exert antioxidant effects evidenced by reductions in blood markers of oxidative modification (16–18), as well as anti-inflammatory properties (17, 19, 20). Nevertheless, compared to exogenous ROS scavengers like vitamin C and E, polyphenols are unlikely to act as direct antioxidants *in vivo* (21). Polyphenol rich supplement consumption, including cherry (22, 23), only results in low plasma phenolic metabolite concentrations relative to urate and other compounds in plasma with antioxidant properties. Instead, evidence suggests that polyphenols upregulate endogenous cellular antioxidant capacity via activation of the Nrf2/ARE pathway (24, 25). Although this hypothesis requires further *in vivo* validation, we previously found that tart cherry supplementation up-regulates gene expression of multiple antioxidant enzymes which are Nrf2 transcriptional targets, and increased glutathione peroxidase (GPX3) protein expression in human skeletal muscle following 7-days of supplementation prior to a bout of intensive exercise and 48 h recovery (23). Some limited evidence also suggests that polyphenols may exert anti-inflammatory effects by inhibiting the activation of the nuclear factor-κ B (NF-κB) (26). NF-kB is a transcription factor that modulates the expression of over 200 genes involved in the body's inflammatory response, including COX-2 and various pro-inflammatory cytokines such as IL-1, IL-6, and TNF-α. Research in rodents found that polyphenol supplementation provided from 2 h post-contusion injury and throughout up to 14d recovery (20 mg.kg^−1^.day of grape seed extract, equivalent to 140 mg.d^−1^ dose for human participants), accelerated muscle ultrastructural recovery, satellite cell response and myosin heavy chain expression (27). Furthermore, in the same study polyphenol supplementation altered immune cell infiltration into the muscle post-contusion, more specifically, the intervention blunted the neutrophil response and accelerated macrophage infiltration in the injured area (28). These data indicate that polyphenol supplementation post-contusion may enhance muscle tissue regeneration and alter the ratio of pro-inflammatory and anti-inflammatory processes within skeletal muscle. Nevertheless, these mechanisms of action require validation via human studies utilizing an exercise-induced muscle damage model with molecular analysis of the damaged skeletal muscle.

To date, multiple studies have found that tart cherry supplementation accelerates functional recovery following a range of exercise modalities including strenuous resistance training (18, 28), endurance running (17, 29) and intermittent running/sprinting protocols (19, 30, 31). The improvements in subsequent performance identified in these studies were found to occur in tandem with a reduction in muscle pain (28, 29), muscle soreness (30) and a decrease in serum markers of oxidative stress (17–19) and inflammation (17, 30). However, the findings are not consistent, with several trials failing to identify an improvement in post-exercise functional recovery following tart cherry supplementation (32–38). These mixed findings are likely due to differences in the supplementation protocol utilized, particularly a lower and ineffective daily polyphenol dose [733 mg and 600 mg, respectively, of total phenolic compounds (32, 37) and a shorter supplementation period (3 days in total with 1 day pre-load and 5 days in total with 2 days pre-load, respectively] (35, 36), as well as utilizing an exercise modality that failed to induce sufficient damage (33, 34, 38). Dose response studies are required to establish an optimal tart cherry supplementation protocol, both in terms of the dose provided and the duration of the supplementation protocol. Furthermore, to date, the antioxidant and anti-inflammatory effects of tart cherries have been explored predominantly through plasma biomarkers that provide a poor surrogate for direct muscle tissue analysis. Blood biomarkers oversimplify the complexity of redox regulation and do not provide a true reflection of the complex interplay of inflammatory processes taking place within the muscle (39, 40). Lastly, the anti-inflammatory and antioxidant properties of tart cherry have been assumed to be responsible for facilitating recovery. Nevertheless, the beneficial effects seen on both post-exercise recovery (17, 18, 23) and exercise performance (41, 42) may be driven by other polyphenol-induced events in the exercised muscle. Hypothesis-free molecular interrogation of these events is therefore warranted; global proteomic analysis of skeletal muscle represents one such approach.

The aim of this investigation was to assess the efficacy of low and high dose of tart cherry supplementation on recovery from exercised-induced muscle damage vs. placebo. We also aimed to further interrogate the molecular mechanisms of tart cherry action in fresh rested and damaged muscle using tandem- mass tagged proteomic analysis of skeletal muscle samples. We hypothesized that tart cherry supplementation would induce signaling via Nrf2/ARE pathway, leading to an upregulation of endogenous antioxidant pathways. We further hypothesized that tart cherry supplementation would alter immune cell infiltration into the damaged skeletal muscle and that such changes may be associated with functional recovery in a dose responsive fashion.

## 2 Methods

### 2.1 Study design

The study employed a double-blinded placebo-controlled crossover design in which participants completed two out of the three conditions (low tart cherry supplementation dose—LTC, high tart cherry supplementation dose–HTC, and placebo–PLA) separated by a two-week washout period in line with previous randomized crossover trials (18, 23, 28). Stratified randomization was used to ensure that the groups were matched and counterbalanced for muscle function. This study design was employed in order to minimize any repeated bout effects. The study received ethical approval from the Sport and Health Sciences ethics committee at the University of Exeter and all the testing was carried out in accordance with the guidance set out by the Declaration of Helsinki. The trial was registered with the Clinical Trial Registry (Registration No. NCT04725149) in December 2020.

### 2.2 Participants

Thirty-four healthy male participants (age = 23.3 ± 4.3 years, weight = 75.5 ± 12.1 kg, height = 178.3 ± 7.3 cm, MVC = 281.7 ± 59.8 N) injury and illness-free were recruited to complete the study. Participants completed a screening questionnaire that included questions related to medical history and inclusion/exclusion criteria, followed by the physical activity readiness questionnaire (PARQ) before providing written informed consent. Inclusion criteria were recreationally active men aged 18–40 y with no medical conditions and no recent (last 3 months) lower limb injury and not taking any nutritional supplements exerting lasting ergogenic effects (e.g. creatine). Females were excluded from the study to prevent any confounding interference of the variation in low-grade inflammation through the menstrual cycle (68). Resistance-trained males were excluded due to their familiarity to strenuous exercise, which is likely to have reduced the severity of the muscle damage inflicted through the exercise protocol (43). To this end individuals were excluded from the study if they had completed more than one lower-body resistance exercise session per week over the last 3 months.

Sample size was calculated as 34 participants to provide 80% power of detecting a statistically significant difference (*p* < 0.05); specifically for PLA vs. LTC group a sample size of 14 based on an effect size of 3% (Cohen's D = 0.87); PLA vs. HTC, a sample size of 8 based on an effect size of 6% (Cohen's D = 1.5); and for LTC vs. HTC a samples size of 12 based on an effect size of 3% (Cohen's D = 0.89). Calculations were based on the maximum voluntary isometric contraction (MVC) force recovery data from Bowtell et al. (18) and the anticipation of a curvilinear relationship between dosage and functional effects on performance markers. Stratified allocation of participants to one of the three groups (PLA vs. LTC; PLA vs. HTC; LTC vs. HTC, respectively) was completed based on the maximal MVC value achieved during their familiarization visit to ensure that all groups were evenly matched in muscle function (Table 1). This was achieved by ranking the familiarization MVC value from highest to lowest within each batch of *n* = 5–6 participants and then allocating a similar range of ranks to each group. The trials completed by each participant were counterbalanced for trial order, and leg dominance within each group. Both the investigators and the participants were blinded to the treatment to avoid potential bias.

**Table 1:** Characteristics of participants in each experimental group (mean ± SD).

Characteristic	PLA-LTC (*n* = 14)	PLA-HTC (*n* = 8)	LTC-HTC (*n* = 12)
Age (y)	24.0 ± 5	22.4 ± 5	23.5 ± 2.6
Height (cm)	176.9 ± 7	179.6 ± 8	179.6 ± 7.5
Weight (kg)	75.1 ± 13	71.1 ± 14	79.2 ± 11.3
MVC (N.m)	276.1 ± 67	287.9 ± 68	276.3 ± 52

The drinks were prepared by and provided to the lead investigator by a researcher who had no other involvement in the study. During each trial, participants completed a 10-day supplementation protocol, consuming one 500 ml bottle of the allocated drink each morning. Each HTC and LTC drink contained 60 ml or 30 ml, respectively, of tart cherry concentrate, meanwhile the PLA drinks were made from fruit concentrate with additional carbohydrate added to ensure all three drinks were isoenergetic. The taste and color matched PLA was formulated and produced by PepsiCo (Gatorade R&D, Barrington, IL, USA) for the purpose of this study. Tart cherry concentrate (Anderson Global Group, USA) was stored at −20 °C and defrosted to allow preparation of the drinks immediately prior to each trial. Participants stored their reconstituted drinks in a refrigerator throughout each 10-day supplementation period, and returned the bottles at the end of each 10-day period to assess compliance, which was 100%. Analysis of the phenolic content of the tart cherry concentrate by HPLC (44) and UPLC-MS with UV-VIS detection (45) was completed at the Quadram Institute Bioscience (Norwich, UK) every 3 months for the study duration to test for stability of polyphenol content (raw data presented in Supplementary Material A). The HTC (60 ml tart cherry concentrate) contained: 32.2 mg anthocyanins, 127.2 mg phenolic acids and 0.3 mg procyanidin. The LTC supplement contained half the polyphenol content of the HTC supplement, equating to 16.1 mg anthocyanins, 63.6 mg phenolic acids, 0.15 mg procyanidin, while the PLA drinks contained insignificant levels of polyphenols.

### 2.3 Physical activity and dietary intake

During the first trial participants were asked to maintain their habitual physical activity/exercise habits, but to avoid any strenuous exercise in the 48 h prior to and after the damaging exercise protocol. Participants were provided with a physical activity and food diary to record food consumed and exercise performed over the 10-day supplementation period. Furthermore, participants were also asked to wear an accelerometer (GeneActiv, Activinsights, Kimbolton, UK) on their non-dominant hand wrist for the duration of the trials, which was used to determine their physical activity levels (46). Participants were provided with a list of high polyphenol foods and asked to minimize but not avoid their intake, except that participants were asked to avoid intake of the following foods on days 7–10 of each trial: black and green tea, alcoholic beverages, coffee and dark chocolate. This approach provides ecological validity as opposed to asking participants to fully avoid the consumption of polyphenol rich foods for the duration of the trial (Supplementary Material D). Prior to the second trial, participants were provided with copies of their trial 1 physical activity and food diary and were asked to replicate their activity/exercise habits and their diet. Participants were asked to record their trial 2 diet; and trial 1 and 2 diet records were then visually checked to confirm that the diets were successfully replicated. Prior to the laboratory testing visits, participants fasted for at least 10 h, with the exception of water and the supplement which was consumed 1 h prior to the visit. All laboratory visits took place at the same hour of the day (±2 h) for each participant.

### 2.4 Experimental protocol

**Figure 1:**
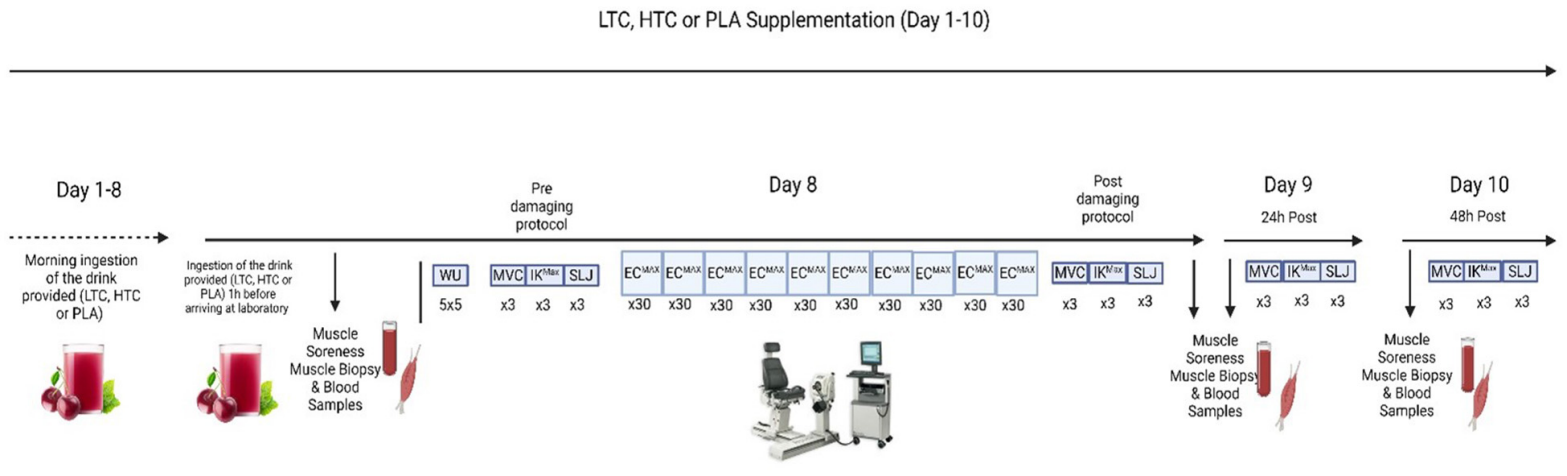
Study Protocol. Visits completed by the participants as part of the experimental design, alongside the main measurements collected. Participants underwent a washout period (minimum 14 days) prior to undergoing a second supplementation condition, with muscle strength, performance and biopsy measurements performed on the opposite leg. WU, warm up; LTC, Low Dose Tart Cherry; HTC, High Dose Tart Cherry; PLA, Placebo; MVC, Maximum Voluntary Contraction; IK^Max^, Maximum Isokinetic Contractions; SLJ, Single Leg Jump; EC^Max^, Maximum Eccentric Contractions.

Following a screening visit where written informed consent was obtained from volunteers, participants returned for a second visit where they were familiarized with all experimental procedures, including the muscle-damaging protocol (10 maximal eccentric knee contractions) and the MVC. Isometric and isokinetic muscle function tests were performed on an isokinetic dynamometer with individualized settings and used to identify leg dominance and muscle strength. Individual dynamometer and chair settings for the isokinetic dynamometer (Biodex Medical Systems, Shirley, USA) were established during this visit and used throughout the participant's remaining study visits. The protocol completed for each trial is presented in Figure 1.

### 2.5 Experimental visits

On day 8 of the supplementation protocol the participants reported in the morning to the laboratory in a fasted state (>10 h since the last meal) having consumed one bottle of tart cherry juice or placebo (500 ml) 1 h prior. Blood samples were collected in tubes containing lithium heparin and were centrifuged at 4,500 rpm for 15 min at 4 °C. Plasma samples were stored at −80 °C in microcentrifuge tubes until analysis. *Vastus lateralis* muscle biopsy samples were taken using the percutaneous suction-modified Bergstrom needle biopsy technique (47). Under local anesthesia and sterile conditions, a small incision (~1 cm) was made through the skin and fascia with a scalpel. The Bergstrom needle was then used under suction to remove a small muscle tissue sample (~100 mg). The wound was then closed with steristrips and dressed with a sterile waterproof dressing. Four biopsies were collected in total for one leg per trial, prior to and after the muscle-damaging protocol, and one at 24 h and 48 h post exercise. The muscle biopsies were collected from separate sites 2–3 cm apart on the same leg within each trial. A portion of each muscle biopsy was snap frozen in liquid nitrogen and stored at −80 °C until analysis. Another portion of each muscle sample (c. 30 mg) was embedded in optimum cutting temperature compound (Tissue-Tek^®^ O.C.T. Compound, Sakura, Alphen aan den Rijn, The Netherlands) and frozen in liquid nitrogen cooled isopentane. Samples were stored at −80 °C until analysis. A urine sample was also collected from the participants and stored at room temperature until the end of the visit (~3 h) when it was used to measure the Urine Specific Gravity (USG) as a measure of hydration and for the Oxidation Reduction Potential (ORP) as a measure of antioxidant capacity.

Muscle soreness measures were completed whilst the participant was seated on the isokinetic dynamometer for the exercised leg. Using a visual analog scale (VAS), subjects rated their level of subjective pain on a 100 mm line. Pressure pain tolerance (PPT) was measured on the belly of *vastus medialis, vastus lateralis*, and *rectus femoris* of the exercised leg using an algometer (FDX 50, Wagner, Greenwich, CT 269 06836-1217 USA). For the algometer measures, pressure exerted was increased at a constant rate until the point of participant discomfort but not pain.

Participants then completed a warm up consisting of 5 sets of 5 non-maximal single leg isokinetic knee extension and flexion repetitions separated by 1 min rest, with a torque limit set at 50% of the maximum force recorded from MVC familiarization. Following the warm up protocol, participants completed 3 maximum isometric voluntary contractions (MVC), separated by a 1 min rest period; followed by 3 sets of 3 maximum eccentric/concentric knee extension (IK) repetitions. Three single leg vertical jumps (SLVJ) were then performed on a mat (Jump Mat Pro, SL Electronics Ltd., Cookstown, UK) with at least a 30-s rest period. Jumps were performed with a single leg take-off and two-footed landing to preserve balance upon landing. Following a further 5-min rest period, participants performed the muscle damage protocol consisting of 10 sets of 30 maximal single leg eccentric knee flexion contractions, with each set separated by a period of 1 min. Total work done during the muscle damage protocol was subsequently calculated.

Following the completion of the muscle damaging protocol, the following measures were collected again: muscle soreness (VAS and PPT), muscle function (MVC, IK and SLVJ). These were followed immediately by the collection of blood samples and a muscle biopsy.

Twenty-four and forty-eight hours later, participants returned to the laboratory following an overnight fast (>10 h since the consumption of the last meal) and the consumption of one bottle of tart cherry juice (500 ml) 1 h prior. Blood samples and a muscle biopsy were collected as part of both visits as per the protocol presented above. All muscle biopsies were collected from the same leg as part of each trial. Muscle soreness measurements (VAS and PPT) were then collected, followed by completion of the warm-up protocol and the muscle function tests (MVC, IKC and SLVJ). Following at least a 2-week supplement wash-out period, this protocol was repeated with the functional measures and damage protocol performed using the contralateral leg, in order to minimize any repeated bout effects.

### 2.6 Force recordings

The knee-extension force (Newtons) produced during exercise was sampled with a frequency of 200 Hz and recorded continuously in a PC via an A/D converter (CED 1401power, Cambridge, UK), using Spike2 (version 8) data acquisition software (CED, Cambridge, UK) with 16 bit resolution.

Total work done during the two muscle damaging protocols was determined via calculation of the area under the force by time curve and was normalized to pre-MVC (relative work done):

Relative work = force × time / pre-MVC

MVC force data were analyzed to identify both peak force output and the highest average value over a 1 s period produced during each contraction. The highest value achieved across the three contractions was reported as the MVC values for that given timepoint. MVC force at each time point was normalized to the pre-exercise value for the specific condition.

IKC force data were analyzed by measuring the peak force generated during each of the three sets which included three individual concentric and eccentric contractions. As for MVC, the highest value achieved across the three sets of concentric and eccentric contractions were the reported concentric IKC and eccentric IKC and normalized to the relevant pre-exercise value.

### 2.7 Blood samples analysis

Plasma samples were analyzed for Interleukin-6 (IL-6) using a Human IL-6 Quantikine HS ELISA kit (R&D Systems, Minneapolis, United States) according to the manufacturer's instructions, to assess systemic inflammation.

### 2.8 Plasma phenolic metabolite analysis

Phenolic metabolite analyses were performed at the Bioanalytical Facility, University of East Anglia. Plasma concentrations of protocatechuic acid, 4-hydroxybenzoic acid, hippuric acid, vanillic acid, ferulic acid, and isoferulic acid were quantified using a Waters Xevo TQ-XS tandem mass spectrometer coupled with an Acquity I-class ultra high-pressure liquid chromatography pump (UPLC) system (Waters Corp., Milford, MA, USA). The LC-MS/MS system was operated in negative electrospray ionization (ESI -ve) mode. Identification and quantification were based on multiple reaction monitoring (MRM) of the respective compound-specific precursor to product ion mass to charge (m/z) transitions: protocatechuic acid 152.9 > 109.2, 4-hydroxybenzoic acid 137.0 > 92.9, hippuric acid 178.0 > 134, vanillic acid 167.0 > 151.9, ferulic acid and isoferulic acid 193.0 > 177.9. Chromatographic separation was achieved using a ModusCore C18 reverse phase column (2.1 m x 50 mm, 2.7 μm) (Chromatography direct, Runcorn, UK) maintained at a temperature of 40 °C. Mobile phases A consisted of 1% acetic acid in LCMS grade deionised water with LCMS grade methanol as mobile phase B. The binary gradient program was: 0 min 1% B, 0–1 min 1% B with a linear increase to 45% B at 10 min, 10–10.5 min 95% B and held to 12 min, returned to 1% B at 12.5 min to re-equilibrate with a cycle time of 15 min. Mobile phase flow rate was 0.5 mL per min throughout the run.

Plasma sample extraction procedure was described in Wangdi et al. (23). In brief, 200 μL of plasma, calibration standards (blank plasma spiked with European Pharmacopeia (EP) reference standards obtained from Merck Germany), and plasma quality controls were spiked with 20 μL of internal standard containing ferulic acid-[2H3] (100 nmol.L^−1^) and hippuric acid [13C6] (200 μmol/L) (Toronto Research Chemicals, Ontario, Canada) in 0.1% formic acid (Merck, Germany) into microcentifuge tubes and mixed. To this, 1 mL of methanol was added slowly with gentle mixing, the mixture was then incubated at room temperature for 15 min, followed by centrifugation at 14,000 rpm for 7 min. The supernatant was transferred to borosilicate glass tube and placed in an evaporator to dry under a constant stream of nitrogen at a temperature of 60 °C. To the dried supernatant 200 μL of methanol (Merck, Germany) with 0.1% formic acid was added into each tube and vortex mixed for 30 s, followed by 2.5 mL of ethylacetate (Merck, Germany) and vigorously mixed for 10 min. After centrifugation at 4,000 rpm for 10 min, 2 mL of the ethylacetate in the upper layer was transferred to a fresh set of borosilicate glass tubes and again evaporated to dryness as described above. The dried residue was resuspended in 250 μL of LCMS grade deionised water with 1% acetic acid (Merck, Germany), then vortex mixed followed by centrifugation at 4,000 rpm for 10 min. The final mixture was transferred into polypropylene autosampler vials, 50 μL was injected into the LC-MS/MS for analysis. MassLynx version 4.2 and QuanLynx software (Waters Corp., Milford, MA, USA) were used for system control, data acquisition, baseline integration and peak quantification. Assay performance is summarized in Supplementary Material A.

### 2.9 Muscle sample analysis

#### 2.9.1 Immune cell infiltration in the exercised muscle

This protocol was used to identify macrophage number in sections of skeletal muscle by targeting the surface marker CD206. A separate channel was used for the identification of neutrophils by using the surface marker CD15.

*Vastus lateralis muscle* samples collected from the participants before, immediately after and 24 h and 48 h post the muscle damaging protocol were frozen in pre-cooled methyl-butane in liquid nitrogen. The muscle samples were aligned for transverse sections and sectioned on cryostat at−25 °C at a set thickness of 7 μm. The cryosections were collected on glass slides, air dried and frozen in slide mailers at −80 °C until staining.

#### 2.9.2 Sample immunostaining

Cryosections were fixed in ice-cold acetone for 3 min. Cryosections were encircled with a hydrophobic barrier and washed once with phosphate buffered saline (PBS). A blocking solution was applied to the cryosections (2% w/v BSA, 5% v/v FBS, 0.2% v/v Triton X-100, 0.1% w/v Sodium azide and 2% v/v Goat serum) for 120 min at room temperature. Cryosections were washed briefly in PBS. Samples were incubated in primary antibodies overnight at 4 °C. Primary antibodies used were: 1) anti-CD206 (deposited to the Developmental Studies Hybridoma Bank DSHB by Stahl, Philip; DSHB Hybridoma Product MR Mab #15; mouse MIgG1), diluted 1:12 in blocking buffer; 2) anti-CD15 (deposited to the DSHB by Solter, D. / Knowles, B.B, DSHB Hybridoma Product MC-480; mouse MIgM, kappa light chain), diluted 1:15 in blocking buffer. Cryosections were washed three times with PBS and then incubated in secondary antibodies for 2 h at room temperature. Secondary antibodies used were: 1) Goat anti-Mouse IgG1 gamma-chain (Alexa Fluor™ 594, Thermo Fisher Scientific, Loughborough, United Kingdom), 2.5 μL/mL in PBS; and 2) Goat Anti-Mouse IgM mu chain (Alexa Fluor^®^ 647, Abcam, Cambridge, United Kingdom), 2.5 μL/mL in PBS. Wheat germ agglutinin (WGA) labeling was used in combination with the secondary antibody at a concentration of 5 μL/mL in PBS. Cryosections were washed three times with PBS and kept at room temperature until dry. A drop of mountant (ProLong Diamond Antifade, Thermo Fisher) was added to each section and a coverslip applied. The cryosections were stored at −80 °C until imaging.

#### 2.9.3 Cryosection imaging

Five images were used to train the Aivia (v12.1.0, Leica Microsystems, Seattle, WA, USA) pixel classifier. Images were processed through an object classifier, with settings designed to identify a fiber number (Min Object Size: 1,000, Max Object Size: 40,000, Background Removal Factor: 100, Contrast Threshold: 0, Fill Holes Size (Remove Background): 0, Smoothing Factor (Remove Background): 0, Intensity Threshold (Skip Remove Background): 0.00305, Fill Holes Size (Skip Remove Background): 500, Smoothing Factor (Skip Remove Background): 10, Separation Factor (Cell Partition): 40 and CD15 positive cells (Min Object Size: 10, Max Object Size: 700, Background Removal Factor: 100, Contrast Threshold: 0, Fill Holes Size (Remove Background): 0, Smoothing Factor (Remove Background): 0, Intensity Threshold (Skip Remove Background): 0.00122, Fill Holes Size (Skip Remove Background): 1, Smoothing Factor (Skip Remove Background): 1, Separation Factor (Cell Partition): 70). Object classifier parameters were adjusted until the fidelity of AI and manual CD15 and fiber number counts was within 5% of the manual counting judgements of the operator. Fiber number and CD15+ cell counts were quantified in two sections per biopsy. For CD206, the object classifier could not be adjusted to achieve acceptable agreement with manual counts. Therefore CD206+ cells were quantified using the ImageJ (release 1.54h) multipoint tool. Four individuals completed these counts. Counters first completed a benchmarking exercise and were blinded to condition and were assigned images in a manner that was counterbalanced for supplement condition and ensured that images from each participant were processed by two independent counters.

#### 2.9.4 Muscle proteomic analysis

*Vastus lateralis muscle* samples from eight individuals who were randomized to receive the high dose of tart cherry product or a placebo for 10 days were used for this analysis. The samples collected immediately before and 48 h after the damaging exercise protocol were analyzed.

#### 2.9.5 Proteomic sample analysis pipeline

Skeletal muscle (15 mg) was placed in microcentrifuge tubes with 250 μL of radioimmunoprecipitation assay (RIPA) buffer (Pierce 89900 RIPA buffer, ThermoFisher Scientific) containing protease and phosphatase inhibitors (Pierce A32961 Protease and Phosphatase Inhibitor EDTA-free mini tablet, ThermoFisher Scientific). Samples were homogenized for 1 min using a bead homogeniser (Speedmill Plus, Analytik Jena AG). Samples were removed to clean microcentrifuge tubes and vortexed thoroughly before incubation on ice for 30 min, with occasional vortexing. Samples were centrifuged for 10 min at 8,000 g at 4 °C. The supernatant was removed to a clean microcentrifuge tube and the pellet was discarded. Protein concentrations were determined by bicinchoninic acid (BCA) assay (Pierce 23225 BCA Protein Assay Kit, ThermoFisher Scientific) according to the manufacturer's instructions.

#### 2.9.6 TMT labeling, high pH reversed-phase chromatography

This work was conducted at the University of Bristol proteomics facility. Aliquots of 50 μg of each sample were digested with trypsin (1.25 μg trypsin; 37 °C, overnight), labeled with Tandem Mass Tag (TMTpro) reagents according to the manufacturer's protocol (Thermo Fisher Scientific, Loughborough, LE11 5RG, UK) and the labeled samples pooled.

An aliquot of 200 ug of the pooled sample was desalted using a SepPak cartridge according to the manufacturer's instructions (Waters, Milford, Massachusetts, USA). Eluate from the SepPak cartridge was evaporated to dryness and resuspended in buffer A (20 mM ammonium hydroxide, pH 10) prior to fractionation by high pH reversed-phase chromatography using an Ultimate 3000 liquid chromatography system (Thermo Fisher Scientific). In brief, the sample was loaded onto an XBridge BEH C18 Column (130 Å, 3.5 μm, 2.1 mm X 150 mm, Waters, UK) in buffer A and peptides eluted with an increasing gradient of buffer B (20 mM Ammonium Hydroxide in acetonitrile, pH 10) from 0–95% over 60 min. The resulting fractions (20 in total) were evaporated to dryness and resuspended in 1% formic acid prior to analysis by nano-LC MSMS using an Orbitrap Fusion Lumos mass spectrometer (Thermo Scientific).

#### 2.9.7 Nano-LC mass spectrometry

High pH RP fractions were further fractionated using an Ultimate 3000 nano-LC system in line with an Orbitrap Fusion Lumos mass spectrometer (Thermo Scientific). In brief, peptides in 1% (vol/vol) formic acid were injected onto an Acclaim PepMap C18 nano-trap column (Thermo Scientific). After washing with 0.5% (vol/vol) acetonitrile 0.1% (vol/vol) formic acid peptides were resolved on a 250 mm × 75 μm Acclaim PepMap C18 reverse phase analytical column (Thermo Scientific) over a 150 min organic gradient, using 7 gradient segments (1–6% solvent B over 1 min, 6–15% B over 58 min, 15–32%B over 58 min, 32–40%B over 5 min, 40–90%B over 1 min, held at 90%B for 6 min and then reduced to 1%B over 1 min) with a flow rate of 300 nl min−1. Solvent A was 0.1% formic acid and Solvent B was aqueous 80% acetonitrile in 0.1% formic acid. Peptides were ionized by nano-electrospray ionization at 2.0 kV using a stainless-steel emitter with an internal diameter of 30 μm (Thermo Scientific) and a capillary temperature of 300 °C.

All spectra were acquired using an Orbitrap Fusion Lumos mass spectrometer controlled by Xcalibur 3.0 software (Thermo Scientific) and operated in data-dependent acquisition mode using an SPS-MS3 workflow. FTMS1 spectra were collected at a resolution of 120 000, with an automatic gain control (AGC) target of 200 000 and a max injection time of 50 ms. Precursors were filtered with an intensity threshold of 5,000, according to charge state (to include charge states 2–7) and with monoisotopic peak determination set to Peptide. Previously interrogated precursors were excluded using a dynamic window (60 s +/– 10 ppm). The MS2 precursors were isolated with a quadrupole isolation window of 0.7 m/z. ITMS2 spectra were collected with an AGC target of 10 000, max injection time of 70 ms and CID collision energy of 35%.

For FTMS3 analysis, the Orbitrap was operated at 50 000 resolution with an AGC target of 50 000 and a max injection time of 105ms. Precursors were fragmented by high energy collision dissociation (HCD) at a normalized collision energy of 60% to ensure maximal TMT reporter ion yield. Synchronous Precursor Selection (SPS) was enabled to include up to 10 MS2 fragment ions in the FTMS3 scan.

#### 2.9.8 Data analysis

The raw data files were processed and quantified using Proteome Discoverer software v2.4 (Thermo Scientific) and searched against the UniProt Human database (downloaded January 2023: 81579 entries) using the SEQUEST HT algorithm. Peptide precursor mass tolerance was set at 10 ppm, and MS/MS tolerance was set at 0.6 Da. Search criteria included oxidation of methionine (+15.995 Da), acetylation of the protein N-terminus (+42.011 Da) and Methionine loss plus acetylation of the protein N-terminus (−89.03 Da) as variable modifications and carbamidomethylation of cysteine (+57.0214) and the addition of the TMTpro mass tag (+304.207) to peptide N-termini and lysine as fixed modifications. Searches were performed with full tryptic digestion and a maximum of 2 missed cleavages were allowed. The reverse database search option was enabled and all data was filtered to satisfy false discovery rate (FDR) of 5%.

#### 2.9.9 Bioinformatics

All proteomic data analyses that follow were conducted at the University of Exeter.

Data were normalized to the total peptide amount in each sample and scaled using a pooled “reference” sample common to both TMT experiments to facilitate the comparison of protein levels between experiments. Data were log2 transformed prior to analysis. Data were filtered to include only proteins that were detected in all samples (3,412 proteins).

#### 2.9.10 Differential protein expression analyses

Differential protein expression analyses were conducted using Reactome's *Correlation Adjusted MEan RAnk* gene set test (CAMERA; reactome.org). Analyses were performed separately on pre-exercise and post-exercise samples. In addition, we examined the main effect of supplement while including exercise as a covariate, in order to provide the broadest possible mechanistic signposting.

#### 2.9.11 Over-representation analysis

A linear mixed model was used to determine the main effects of supplement, time and their interaction on individual protein expression (3,412 proteins). Analyses were implemented in R using the lmerTest package, with *post-hoc* Hochberg corrections applied to account for multiple comparisons.

#### 2.9.12 STRING protein-protein interaction analysis

The proteome was visualized in Cytoscape v3.10.0 using stringApp v2.0.1. The full STRING network was used with a high confidence threshold of 0.7. Log2 fold change values were used to style the network, representing differential expression in the cherry condition relative to placebo. Network clustering was performed using the Markov clustering (MCL) implementation in the clusterMaker2 Cytoscape app with an inflation value of 4.0.

##### 2.9.12.1 Correlation analyses with pathway enrichment scores

Exploratory correlation analyses were conducted to examine associations between plasma phenolic metabolites, skeletal muscle functional measures, immune cell infiltration, plasma inflammatory markers, and pathway-level enrichment. Pathway activity was estimated using Reactome single-sample gene set enrichment analysis (ssGSEA) scores, providing participant-level resolution. Pathways included in the analysis were selected based on parent pathways found to be altered in at least one standalone analysis.

To further explore relationships between individual plasma phenolic metabolites and pathway-level proteomic activity, linear mixed-effects models (LMMs) were employed. These models accounted for the repeated-measures structure of the data (multiple samples per participant) by including a random intercept for each participant. Separate LMMs were fitted for each phenolic metabolite and each selected pathway ssGSEA score as the dependent variable. Fixed effects included the metabolite concentration of interest, Supplement condition (Placebo or Supplement), and Exercise status (Rest or Exercise), along with the interaction between Supplement and Exercise. This structure allowed us to estimate the association between each phenolic metabolite and pathway activity while adjusting for potential confounding effects of the intervention and exercise bout.

Models were fit using the lme4 and lmerTest packages in R (v4.3.0), with the restricted maximum likelihood (REML) estimation method and the “bobyqa” optimizer to ensure model convergence. Separate models were run for each combination of phenolic metabolite and pathway, and estimated coefficients, standard errors, *t*-values, and *p*-values for the metabolite term were extracted. Given that pathways were pre-selected based on prior multiple-comparison-adjusted analyses, no additional correction for multiple testing was applied in these targeted LMM analyses.

### 2.10 Statistical analysis

The primary outcome (MVC) and each of the secondary outcomes were analyzed separately using “mixed effects regression modelling” to assess differences between the conditions (PLA, LTC with 30 ml TC concentrate in a 500 ml beverage, HTC with 60 ml TC concentrate in a 500 ml beverage). This approach is appropriate for this mixed model design since paired and non-paired data are used to estimate differences between conditions. Condition was entered into the mixed effects model as both a fixed effect and a random effect. The mixed effects regression model contained parameter estimates coefficients (with 95% confidence intervals and *p* values) for two of the three levels LTC and HTC reflecting the estimated difference from the reference level (PLA). This enabled comparison of PLA vs. LTC vs. HTC. The model was then re-run with HTC as the reference level to test the remaining comparison of LTC vs. HTC. Adjustment for multiple comparisons was not necessary as effectively only one model, with one factor, was produced. *P* < 0.05 (2-sided) was considered statistically significant. Histograms of the model residuals were used to check for normality and any outliers. All statistical analyses were carried out using SPSS version 29.

**Figure 2:**
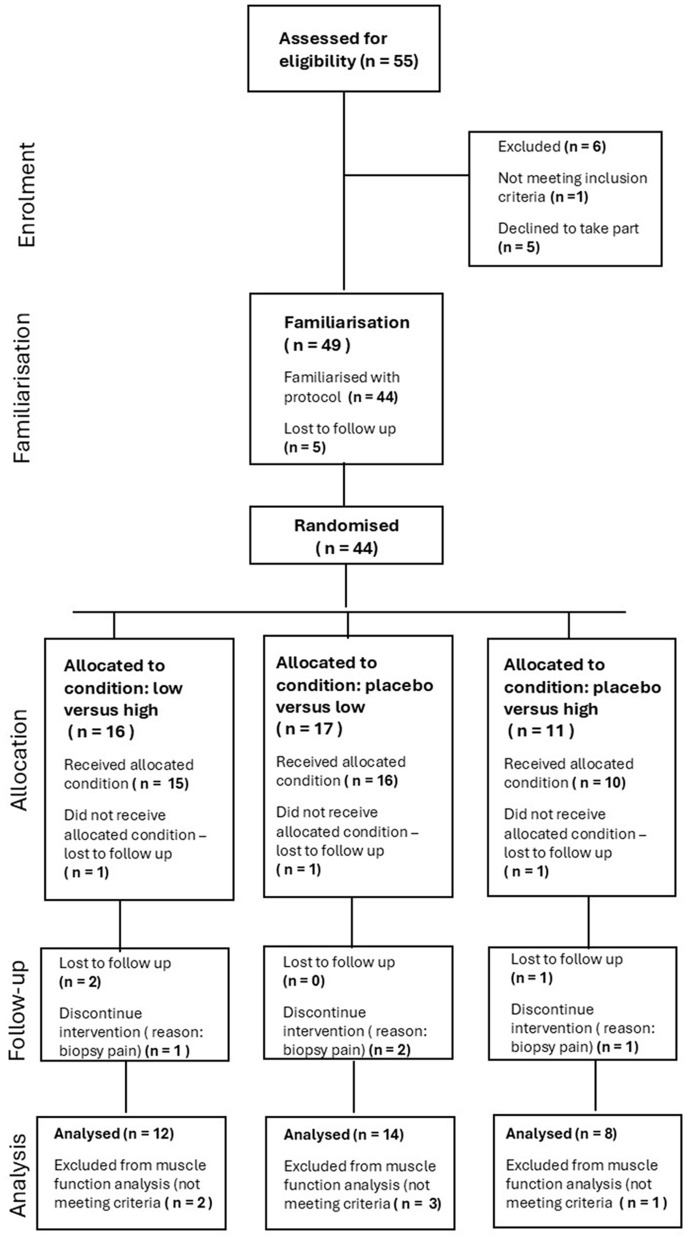
Consort Diagram indicating participants recruitment and study completion.

Prior to unblinding, the MVC and isokinetic functional data for all participants were screened using the following criteria to check for full volitional effort during the damaging protocol and for the performance measures: (i) > 20% difference in relative work done during muscle damage exercise protocol between trials; (ii) > 20% difference between trials in magnitude of MVC reduction immediately post damaging protocol and (iii) MVC after 48 h recovery > 110% of baseline MVC. On this basis, four participants were excluded from the statistical analysis for the muscle function outcomes due to differences in the relative work done during the muscle damage protocol (22–67% difference between trials, >2SD from the group mean) resulting in different magnitudes of force reduction immediately post exercise between the two trials. Two participants were excluded from the statistical analysis for the muscle function outcomes as their isometric strength (MVC) after 48 h of recovery reached 110% of baseline MVC, indicating that a true baseline was not measured thus confounding comparisons between trials. The progression of the participants and their inclusion in the statistical analysis is presented in the Consort Diagram in Figure 2.

## 3 Results

### 3.1 Muscle function

There was no difference between the three trials (PLA, LTC and HTC) in the amount of relative work done by subjects during the muscle-damaging protocol (185 ± 29; 196 ± 46; 203 ± 45 relative units; p =0.697).

**Figure 3:**
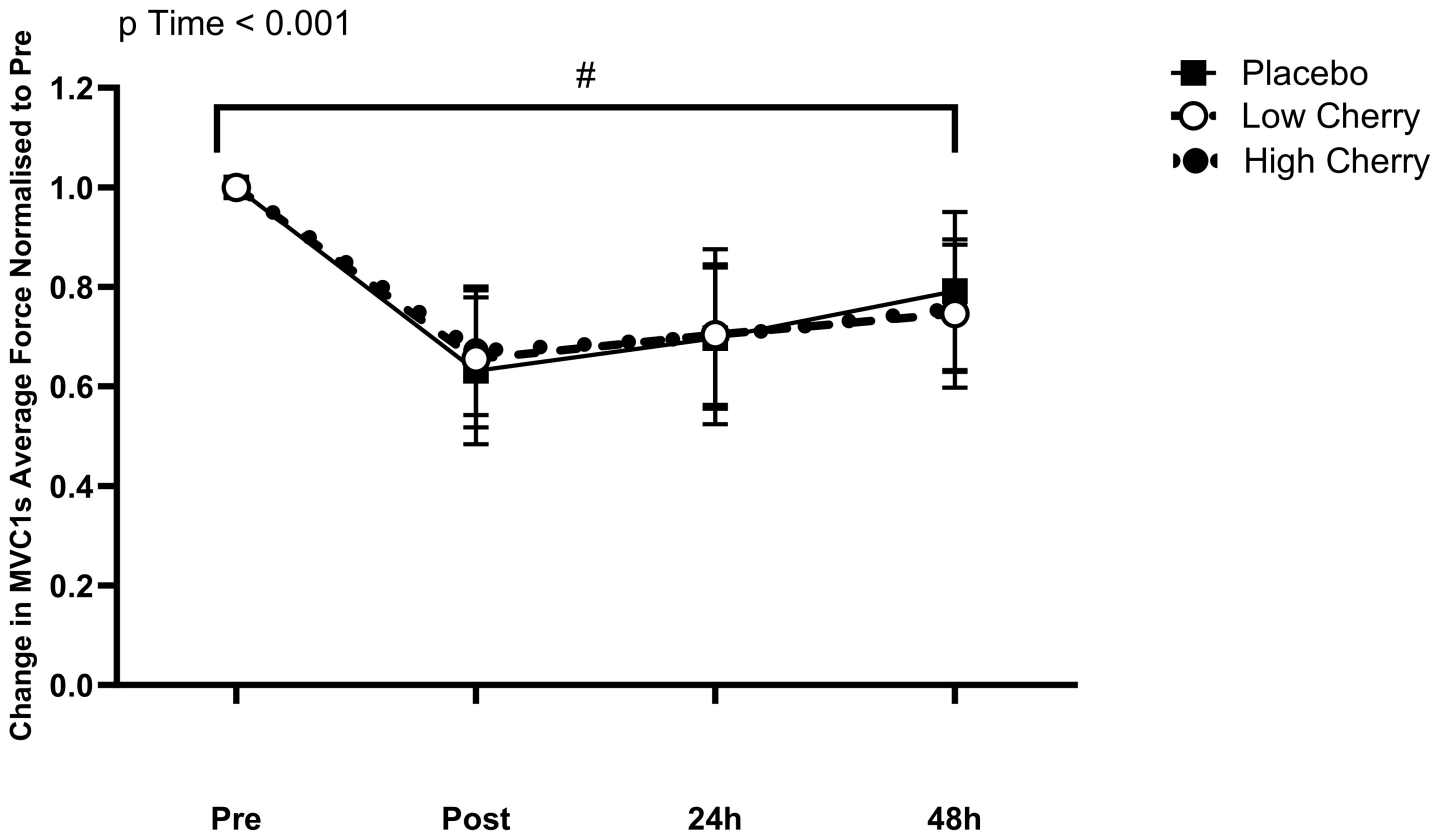
MVC1s average force normalized to pre-exercise values at the following timepoints: pre-damaging protocol, immediately after, 24 h and 48 h after the damaging protocol; # denotes a significant main effect for time (*p* < 0.05). Values are presented as means ± SD.

Normalized knee extension MVC 1 s average force decreased to 0.63 ± 0.13, 0.65 ± 0.14 and 0.67 ± 0.13 of pre-exercise levels for LTC, PLA and HTC, respectively, following the completion of the muscle-damaging protocol (main effect of time, *p* < 0.001). Recovery of normalized MVC 1s average was not different between the three conditions (24 h post: 0.70 ± 0.14, 0.70 ± 0.14 and 0.70 ± 0.17; 48h post: 0.79 ± 0.15, 0.74 ± 0.14 and 0.76 ± 0.12 for PLA, LTC, and HTC respectively; condition effect, *p* = 0.067, interaction effect, *p* = 0.279; Figure 3). Pre-exercise MVC 1 s average force before the damaging protocol was 272.8 ± 53.8 N, 275.6 ± 60.3 N and 272.6 ± 61.1 N for PLA, LTC, and HTC, respectively.

**Figure 4:**
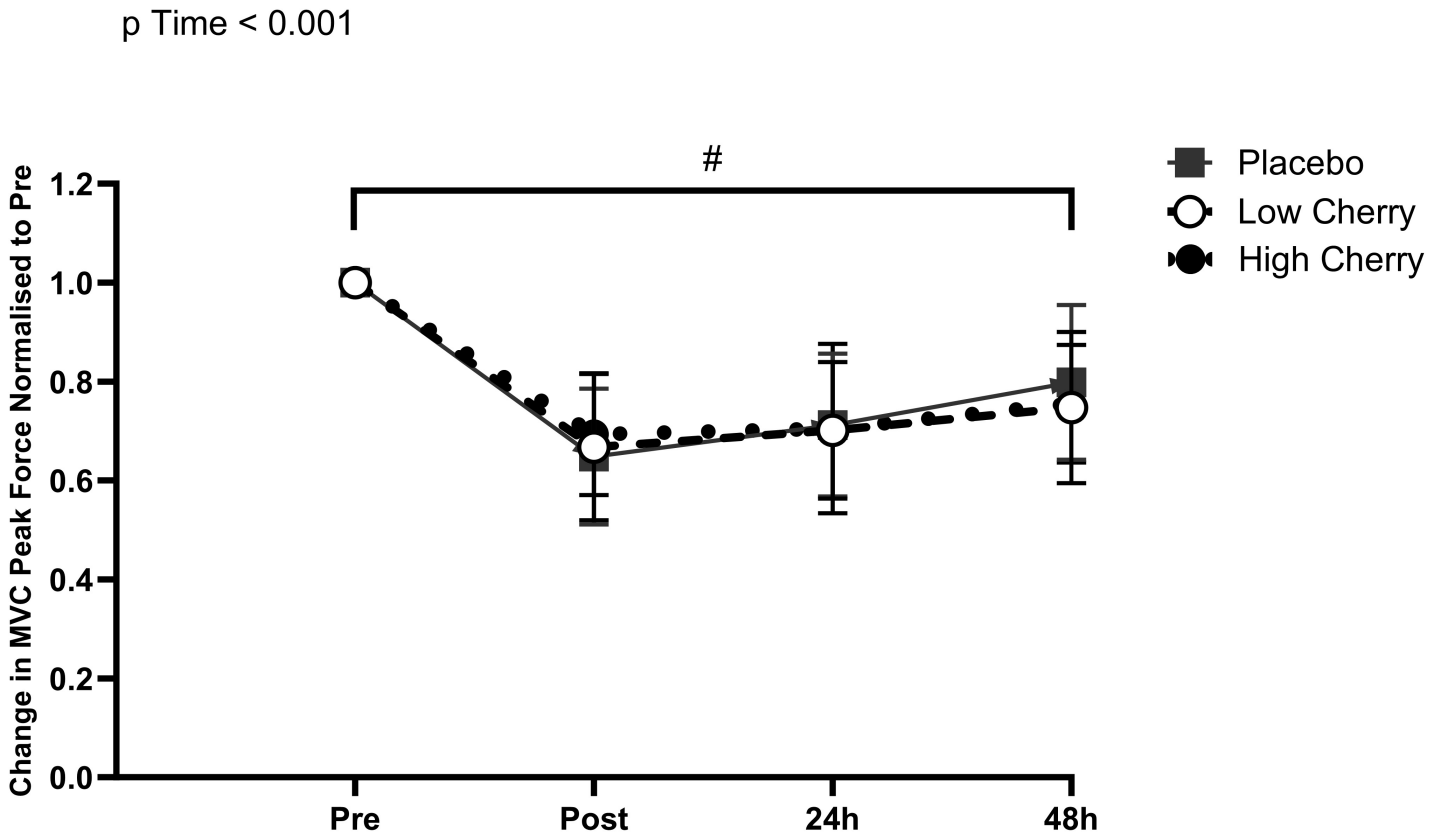
MVC peak force normalized to pre-exercise values at the following timepoints: pre-damaging protocol, immediately after, 24 h and 48 h after the damaging protocol; # denotes a significant main effect for time (*p* < 0.05). Values are presented as means ± SD.

Normalized knee extension MVC peak force decreased to 0.65 ± 0.13, 0.67 ± 0.14 and 0.69 ± 0.12 of pre-exercise levels for PLA, LTC, and HTC respectively, following the completion of the muscle-damaging protocol (main effect of time, *p* < 0.001). Recovery of normalized MVC peak was not different between the three conditions (condition effect, *p* = 0.085, interaction effect, *p* = 0.279) (Figure 4, see Supplementary Material B for non-normalized data). MVC peak returned to 0.71 ± 0.14, 0.70 ± 0.13, and 0.70 ± 0.17 at 24 h post for PLA, LTC and HTC respectively, and to 0.80 ± 0.15, 0.75 ± 0.15 and 0.76 ± 0.12 at 48 h post. Pre-exercise peak MVC force before the damaging protocol were 279.7 ± 53.5 N, 282.3 ± 57.8 N and 281.3 ± 62.6 N for LTC, PLA, and HTC, respectively.

**Figure 5:**
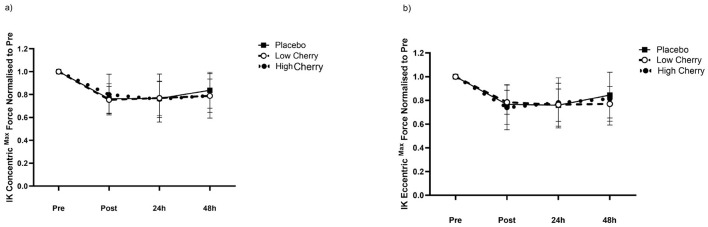
**(A)** Peak concentric phase maximal isokinetic contractions. **(B)** Peak eccentric phase maximal isokinetic contractions. Values are presented as means ± SD.

There was no significant difference between the trials in recovery of normalized IK^Max^ for either the concentric (IK Concentric ^Max^) (main effect of condition, *p* = 0.056) (Figure 5A) or the eccentric (IK Eccentric ^Max^) contraction phase (main effect of condition, *p* = 0.231) (Figure 5B). There was also no significant difference between the trials in recovery of jump height (*p* = 0.353) (Table 1).

There was a significant increase in VAS after the muscle-damaging protocol (main effect of time, *p* < 0.001) indicating the development of muscle soreness (Table 2). There was no difference between the trials in VAS (main effect of condition, *p* = 0.800). There was also a significant decrease in PPT in the three muscles tested (VL, VM, and RF) (main effect of time, *p* < 0.001) (Table 1), also indicating an increase in muscle soreness following the damaging protocol. There was no difference between the trials in PPT^VL^ (condition effect, *p* = 0.191) and PPT^RF^ (condition effect, *p* = 0.507), however, a significant effect was seen for condition (*p* = 0.020) on PPT^VM^, with a lower reduction in PLA compared to LTC (*p* = 0.002).

**Table 2:** Visual analog scale (VAS) and Pressure Pain Tolerance for the *Vastus Lateralis* (VL), *Vastus Medialis* (VM) and *Rector Femoris* (RF) muscles in the three conditions (PLA, LTC and HTC) at the following timepoints: pre-damaging protocol, immediately after, 24 h and 48 h after the damaging protocol.

Measure	Supplement	Pre-exercise	Post-exercise	24 h	48 h	Condition Effect (*P*)	Time Effect (*P*)
Jump (m)	PLA	0.27 ± 0.06	0.20 ± 0.10	0.23 ± 0.06	0.23 ± 0.07	0.353	0.376
	LTC	0.27 ± 0.05	0.22 ± 0.04	0.23 ± 0.04	0.23 ± 0.04		
	HTC	0.28 ± 0.05	0.22 ± 0.08	0.24 ± 0.07	0.24 ± 0.05		
VSA (mm)	PLA	6.3 ± 11.5	28.6 ± 26.7	49.6 ± 14.1	55.8 ± 17.9	0.800	< 0.001
	LTC	6.2 ± 10.7	17.9 ± 17.9	51.7 ± 22.5	55.0 ± 27.2		
	HTC	5.6 ± 7.3	14.7 ± 13.6	51.8 ± 19.8	56.8 ± 23.9		
PPT^VL^ (N)	PLA	85 ± 19.3	86 ± 17.1	63. ± 23.0	58 ± 21.7	0.191	< 0.001
	LTC	81 ± 15.4	77. ± 15.5	61 ± 11.4	59 ± 21.2		
	HTC	81 ± 25.1	79 ± 22.8	59 ± 16.8	62 ± 21.2		
PPT^Vm^ (N)	PLA	79 ± 14.6	76 ± 15.4	55 ± 18.5	54 ± 20.5	0.020	< 0.001
	LTC	76 ± 17.3	73 ± 17.1	49 ± 16.4	45 ± 15.3		
	HTC	70 ± 15.2	71 ± 18.6	55 ± 27.5	46 ± 16.1		
PPT^RF^ (N)	PLA	111 ± 17.3	116 ± 14.5	83 ± 25.9	79 ± 25.4	0.507	< 0.001
	LTC	109 ± 19.7	107 ± 30.9	79 ± 28.8	75 ± 29.2		
	HTC	105 ± 28.4	107 ± 30.9	76 ± 26.0	71 ± 23.1		

Values are presented as means ± SD.

### 3.2 Plasma phenolic acid concentrations

**Figure 6:**
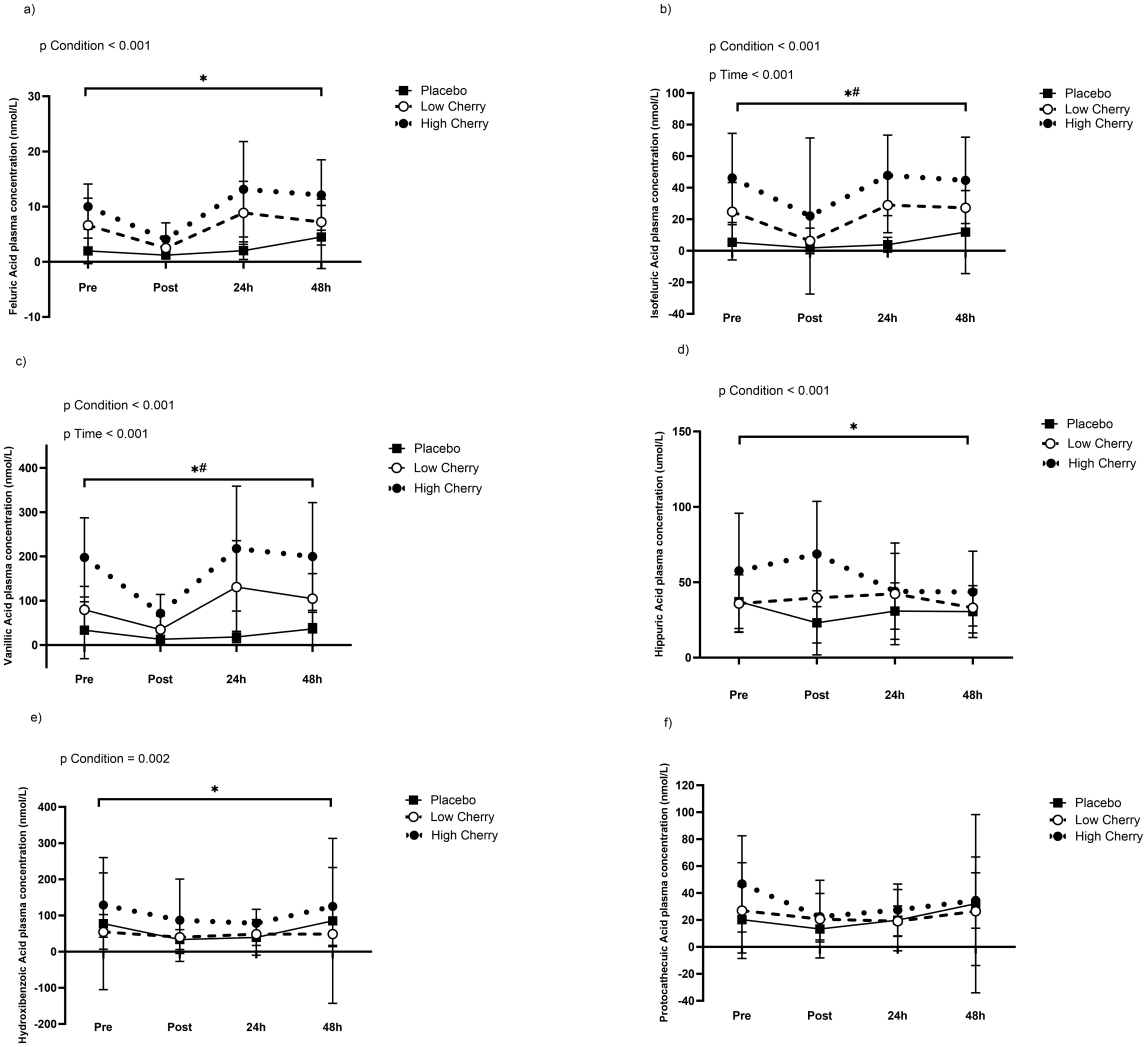
Plasma phenolic acids concentration for the three conditions measured at the following timepoints: pre-damaging protocol, immediately after, 24 h and 48 h after the damaging protocol. Metabolites displayed are **(a)** Ferulic Acid, **(b)** Isoferulic Acid, **(c)** Vanillic Acid, **(d)** Hippuric Acid, **(e)** Hydroxybenzoic Acid, and **(f)** Protocatechuic Acid. Values are presented as means ± SD. # denotes a significant main effect for time (*p* < 0.05); * denotes a significant main effect for condition (*p* < 0.05).

Plasma ferulic acid, isoferulic, and vanillic acid concentrations were increased following both the LTC and HTC compared to PLA (main effect of condition, *p* < 0.001) (Figures 6A–C). For these three phenolic acids, concentrations were also higher during the HTC vs. the LTC trial (*p* < 0.001). There was also a main effect for condition on plasma hippuric (Figure 6D) and hydroxybenzoic acid (Figure 6E) concentrations (*p* < 0.001), with the HTC condition leading to increased concentrations compared to PLA (*p* < 0.001) and LTC (*p* < 0.001) for hippuric acid, and increased concentrations compared to PLA (*p* < 0.001) and LTC (*p* = 0.016) for hydroxybenzoic acid. There was no significant difference between PLA and LTC for plasma concentrations of hippuric acid (*p* = 0.051) and of hydroxybenzoic acid (*p* = 0.357). There was no significant difference between the trials for plasma protocatechuic acid concentrations (main effect of condition, *p* = 0.086) (Figure 6F).

### 3.3 Plasma IL-6 and immune cell infiltration of exercised muscle

**Figure 7:**
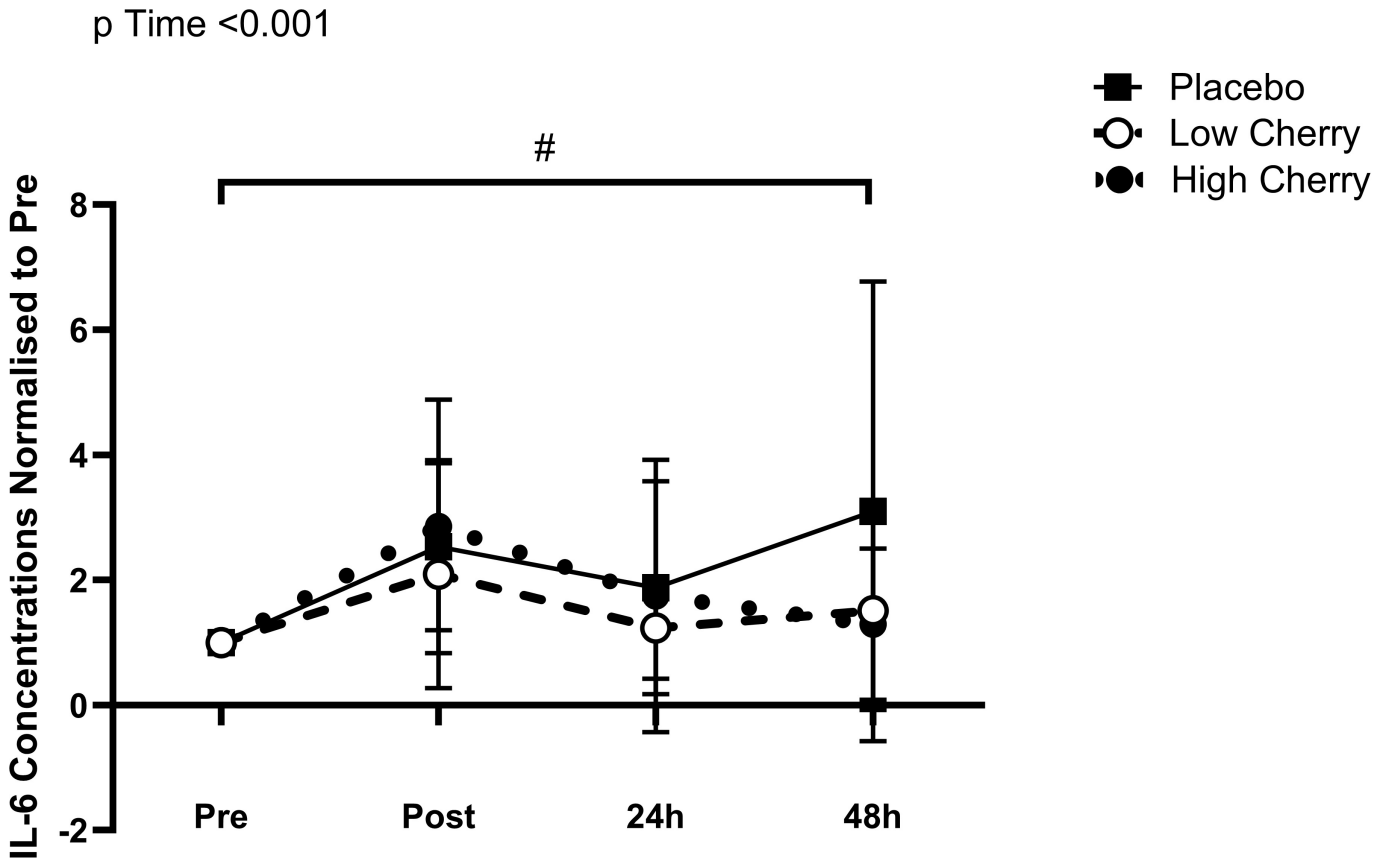
Normalized plasma IL-6 concentrations to pre-exercise measured at the following timepoints: pre-damaging protocol, immediately after, 24 h and 48 h after the damaging protocol; # denotes a significant main effect for time (*p* < 0.05). Values are presented as means ± SD.

There was a significant increase in normalized plasma IL-6 concentrations from pre-exercise following the muscle-damaging protocol (main effect of time, *p* < 0.001), indicating an increase in systemic inflammation (Figure 7). However, there was no difference between the trials in normalized IL-6 concentrations (main effect of condition, *p* = 0.103).

**Figure 8:**
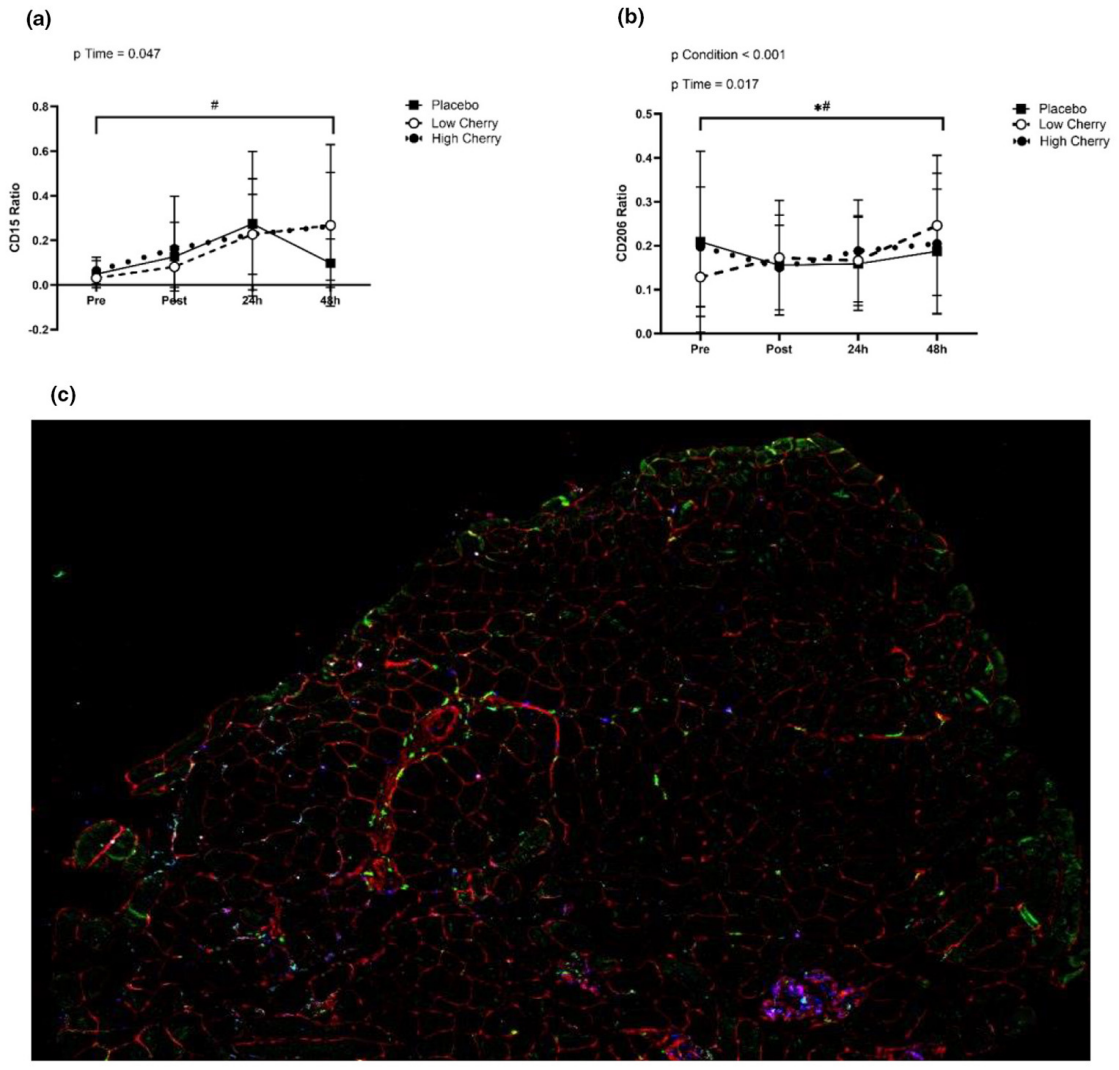
**(a)** Figure displaying the CD15 number to muscle fibers ratio. **(b)** Figure displaying the CD206 number to muscle fibers ratio. Both CD15 and CD206 were measured at the following timepoints: pre-damaging protocol, immediately after, 24 h and 48 h after the damaging protocol. # denotes a significant main effect for time (*p* < 0.05); * denotes a significant main effect for condition (*p* < 0.05). Values are presented as means ± SD. **(c)** Picture displaying an image of a muscle section.

There was an increase in CD15 to fiber ratio following the muscle-damaging protocol (main effect of time, *p* = 0.047) (Figure 8A). However, there was no difference between the trials in CD15 to fiber ratio (main effect of condition, *p* = 0.857). A main effect for time was also seen in the CD206 to fiber ratio following the muscle-damaging protocol (*p* = 0.017) (Figure 8B). There was also a significant difference between trials (main effect of condition, *p* < 0.001), with an increased CD206 to fiber ratio in the LTC condition compared to PLA (*p* < 0.001) and compared to HTC (*p* = 0.009).

### 3.4 Skeletal muscle proteomics

**Figure 9:**
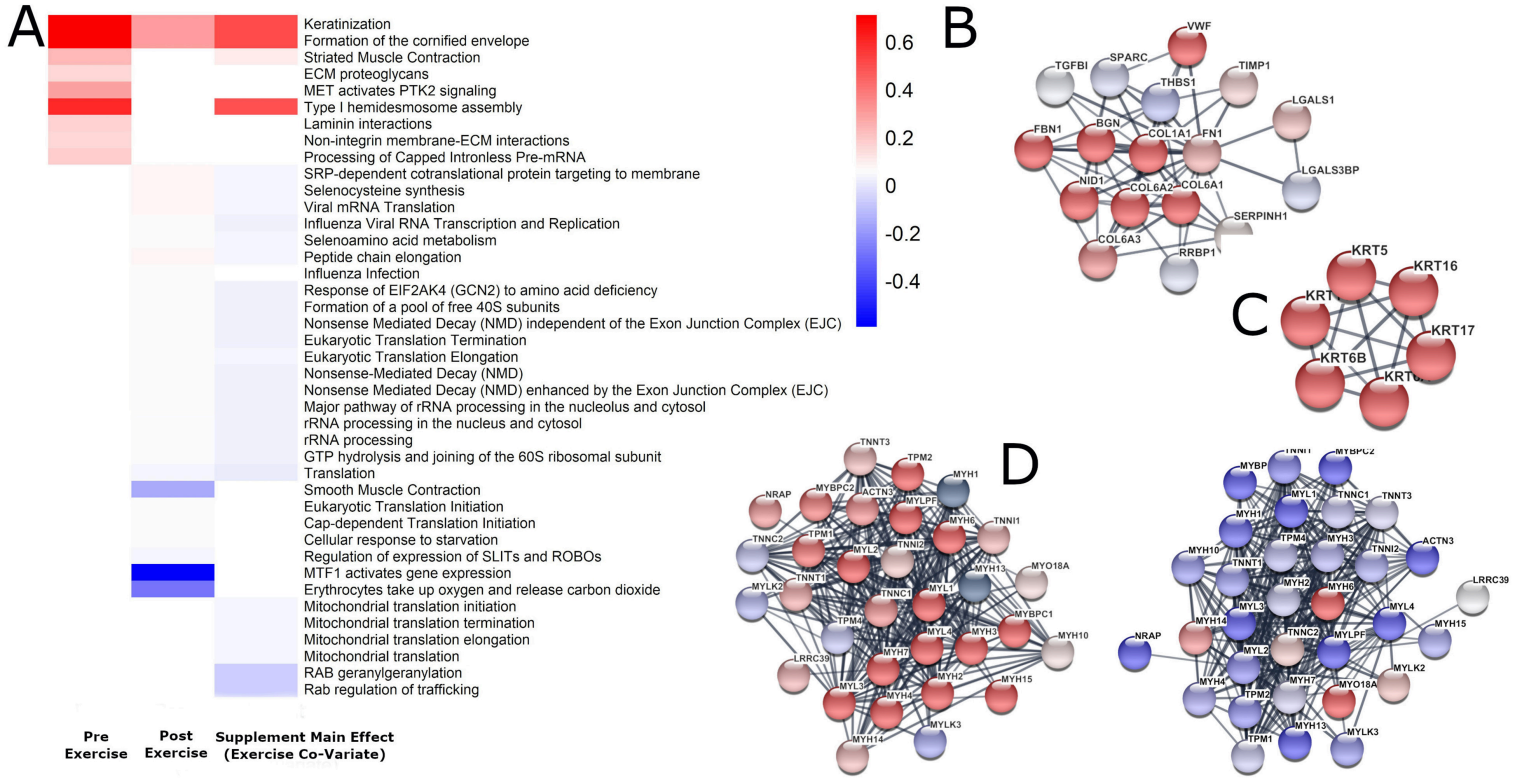
Visualization of skeletal muscle protein pathways most prominently altered by tart cherry supplementation before and after (48 h) damaging resistance exercise. **(A)** Reactome pathway themes significantly altered by tart cherry supplementation. A blue–white–red gradient was styled using the mean log_2_ fold change value for each theme compared to placebo values both pre and post damaging exercise; for completeness, results of a Reactome analysis of main effect of supplementation with exercise as a covariate in the analysis are also presented **(B–D)** To provide visual corroboration of Rectome analyses, Log_2_ fold changes between placebo and cherry supplemented groups for each protein were mapped to nodes using a blue–white–red gradient. To aid readability, protein names are indicated by their official gene symbol. **(B)** Extracellular matrix proteins, **(C)** Keratins, and **(D)** Muscle contractile proteins.

Reactome analyses indicate that 7 days of tart cherry supplementation increased the expression of muscle contractile proteins (Figures 9A, D). Following damaging exercise, tart cherry supplementation reduced the expression of muscle contractile proteins (Figure 9D). We observed a cherry-induced increase in pre-exercise levels of skeletal muscle proteins related to keratinization (intermediate filaments), the extracellular matrix, type 1 hemidesmosomes, and laminin interactions (Figure 9). Proteins related to keratinization (intermediate filaments) remained elevated in the cherry condition post-damaging exercise.

**Figure 10:**
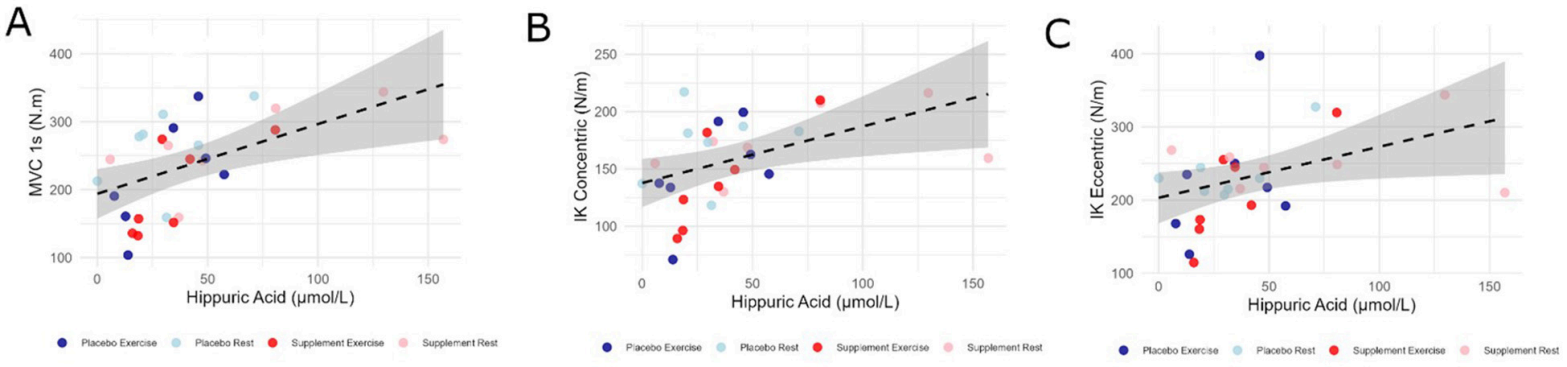
Associations between circulating hippuric acid and skeletal muscle function. Panels show representative relationships between plasma hippuric acid concentrations and measures of skeletal muscle function. Colored points indicate individual participants grouped by Supplement and Exercise condition (Placebo Rest, Placebo Exercise, Supplement Rest, and Supplement Exercise). The black dashed line represents a simple linear trend for visualization purposes only. Higher circulating hippuric acid concentrations were positively associated with maximal voluntary contraction [MVC; **(A)**], isokinetic concentric torque **(B)**, and isokinetic eccentric torque **(C)**. These relationships were observed at all individual testing timepoints (baseline, post-exercise, 24 h, and 48 h) as well as when analyzed overall.

We carried out an exploratory analysis to examine the relationships between phenolics and skeletal muscle functional measures, skeletal muscle pathways (selected based on parent pathways found to change in at least one standalone analysis), in order to identify mechanistic links warranting further investigation. Notably, circulating hippuric acid concentrations (baseline, post-exercise, 24 h and 48 h), but not those of other phenolics were positively correlated with MVC values attained at all individual (baseline, post-exercise, 24 h and 48 h) strength testing timepoints (Pearson r across all MVC 1 s timepoints= 0.56, *p* = 0.004; Figure 10). Circulating hippuric acid concentrations (baseline, post-exercise, 24 h and 48 h) were also significantly and positively correlated with IKconcentric and IKeccentric values at most such timepoints (Figure 10). Notably, pre-exercise hippuric acid concentration was different across conditions (one way ANOVA, main effect of condition, *p* = 0.030). *Post-hoc* tests indicate that pre-exercise plasma hippuric acid was significantly higher for HTC than LTC, but not placebo condition (Figure 6).

**Figure 11:**
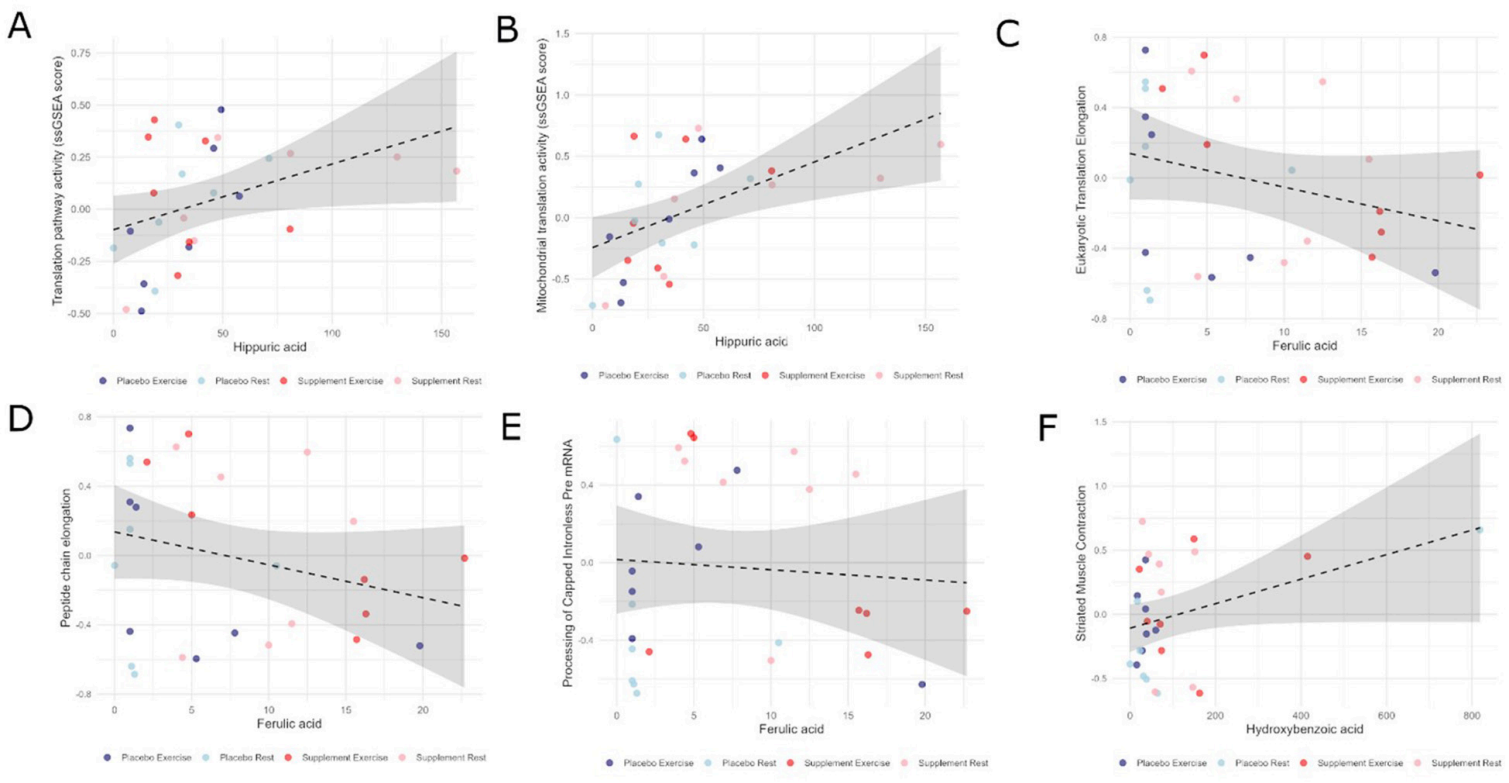
Associations between circulating phenolic metabolites and skeletal muscle proteomic pathway activity. Panels show representative relationships identified by linear mixed models adjusting for supplement and exercise status. Colored points indicate individual participants grouped by Supplement and Exercise condition (Placebo Rest, Placebo Exercise, Supplement Rest, Supplement Exercise). The black dashed line represents a simple linear trend for visualization purposes only; statistical inference was based on linear mixed models (LMMs) with participant-level random intercepts. **(A, B)** Hippuric acid positively associates with mitochondrial and cytosolic translation pathways, including Translation **(A)** and Mitochondrial Translation **(B)**. **(C–E)** Ferulic acid negatively associates with pathways involved in mRNA processing and translation, including Eukaryotic Translation Termination **(C)**, Peptide Chain Elongation **(D)**, and Processing of Capped Intronless Pre-mRNA **(E)**. **(F)** Hydroxybenzoic acid positively associates with Striated Muscle Contraction, suggesting a potential link to contractile function.

Linear mixed model analyses adjusting for supplement and exercise status revealed distinct associations between circulating phenolic metabolites and skeletal muscle pathway activity. Higher plasma hippuric acid concentrations were positively associated with multiple mitochondrial and translational pathways, including Mitochondrial translation (β = 0.0056 ± 0.0021, *p* = 0.016), Mitochondrial translation termination (β = 0.0053 ± 0.0021, *p* = 0.021), Mitochondrial translation elongation (β = 0.0052 ± 0.0021, *p* = 0.023), Mitochondrial translation initiation (β = 0.0054 ± 0.0022, *p* = 0.024), and the global Translation pathway (β = 0.0040 ± 0.0017, *p* = 0.025). These findings suggest that hippurate is positively linked to mitochondrial and cytosolic protein synthesis processes. Trends toward inverse associations were also observed for epidermal differentiation pathways, including Formation of the cornified envelope and Keratinization (both *p* ≈ 0.05).

In contrast, ferulic acid concentrations were inversely associated with several pathways related to mRNA processing, translation, and RNA surveillance. Significant negative relationships were observed for Processing of capped intronless pre-mRNA (β = −0.0367 ± 0.0153, *p* = 0.026), SRP-dependent cotranslational protein targeting to membrane (β = −0.0365 ± 0.0158, *p* = 0.031), Peptide chain elongation (β = −0.0385 ± 0.0171, *p* = 0.034), and Eukaryotic translation termination (β = −0.0356 ± 0.0164, *p* = 0.041). Additional modest inverse associations were detected for Nonsense-mediated decay pathways, rRNA processing, and Selenocysteine synthesis (all *p* < 0.05).

Finally, hydroxybenzoic acid showed a positive association with Striated muscle contraction (β = 0.0011 ± 0.0005, *p* = 0.034), suggesting a potential link between this metabolite and muscle contractile function. Collectively, these relationships indicate that distinct classes of phenolic metabolites may differentially modulate mitochondrial protein synthesis, RNA metabolism, and muscle structure-related proteomic pathways.

## 4 Discussion

Tart cherry supplementation induced a significant elevation in multiple plasma phenolic metabolites in tandem with altered macrophage infiltration in the exercised muscle. This study provides the first evidence that a multi-day tart cherry supplementation protocol including a 7-day pre-load and 3-day post-load leads to differential changes in structural and contractile muscle proteins. After 7 days supplementation, both contractile and structural proteins were increased, however post-exercise whilst intermediate filament proteins remained elevated, contractile proteins decreased in the cherry condition. Further, following exercise, we demonstrate for the first time increases in proteins from pathways related to protein translation in response to cherry supplementation. Uniquely for this field, we carried out an analysis which allowed us to explore relationships between plasma concentrations of phenolic metabolites at different timepoints in relation to the muscle damaging protocol and changes seen in various molecular pathways. Higher plasma hippuric acid levels were positively associated with knee extensor strength and proteins in mitochondrial and translational pathways. Ferulic acid showed inverse associations with mRNA processing and translation pathways. In contrast, hydroxybenzoic acid was positively associated with proteins related to striated muscle contraction. Despite molecular findings which considerably advance this field, tart cherry supplementation did not improve muscle function recovery in the present study.

This is the first study to identify the changes that occur in the muscle proteome in response to a multi-day tart cherry supplementation protocol both prior to and following the completion of intensive muscle-damaging exercise. First, the intensive exercise protocol increased proteins in pathways related to the immune system, in line with previous work investigating immunological changes in skeletal muscle following eccentric exercise (48, 49). This observation provides confidence in the physiological validity of our proteomic data. Despite previous suggestions that tart cherry supplements may exert their functional effects on skeletal muscle via an anti-inflammatory mechanism (17, 19), we found no effect of tart cherry supplementation on any inflammatory pathways in skeletal muscle. Our study is the first to show evidence that tart cherry supplementation leads to an increase in the expression of muscle contractile proteins following a 7-day pre-load protocol. This was followed by a decrease in the quantity of such proteins at 48 h post damage. An increase in contractile proteins in skeletal muscle prior to a damaging stimulus may confer a pre-conditioning effect for skeletal muscle. Indeed, this would fit with the current literature, which has coalesced around the notion that pre-loading with a sufficient cherry dose, for > 5 days is necessary to aid recovery from damage (17, 18, 23). Notably, a separate PADOG analysis of the cherry and placebo proteomes indicated that there was no effect of time i.e., muscle damage on levels of muscle contractile proteins; so exercise does not appear to confound our observations. We hypothesize that pre-conditioning of the skeletal muscle contractile protein pool by tart cherry may reduce the need for such proteins to be expressed post-damage.

We also identified that tart cherry supplementation increased quantities of proteins involved in the mechanical integrity of skeletal muscle (intermediate filaments, extracellular matrix, hemidesmosomes, and laminin interactors). This was particularly prominent pre-exercise after the 7 day pre-load, although keratin/intermediate filament protein expression remained elevated by tart cherry post-damage. Hemidesmosomes are integral in linking the extracellular proteins to the basement membrane and intracellular intermediate filaments (50). The co-ordinated upregulation of this non-contractile protein network by cherry is notable, given that it is increasingly understood that it plays a central role in physical force transfer between the extracellular and intracellular environments (51). These proteins are relatively understudied in skeletal muscle, with much of the literature limited to the study of desmin. It is notable that desmin is necessary for normal nuclear deformation and stress-mediated JNK signaling in response to skeletal muscle stretch (52). Therefore, we suggest that tart cherry supplementation may reinforce this structurally and functionally important protein network prior to muscle damage. Eccentric exercise produces high tensile forces which induces muscle damage (53), so our observations of changes in the structural protein network may contribute to the beneficial effects seen on recovery of force production following damaging exercise in previous studies with tart cherry supplementation (18, 23, 28). We also observed that following damaging exercise, tart cherry supplementation increased expression of proteins in pathways related to protein translation. Enhanced protein translation is thought to be the major determinant of metabolic and physical adaptations in response to exercise training–regardless of modality and participant age (54). Taken together, tart cherry-induced modification of the skeletal muscle proteome characterized here suggests that tart cherry polyphenols may enhance exercise training adaptation. Further studies are however required to elucidate this. The hypothesized link between tart cherry supplementation and longer-term skeletal muscle function is further strengthened by the observation that levels of circulating phenolic acids immediately pre and post damaging exercise displayed a significant positive correlation with enrichment of a number of pathways which may influence muscle adaptive responses. The response to supplementation with tart cherry and other complex natural blends of polyphenols is unlikely to depend on single metabolites but instead reflect an integrated response to a highly complex blend of bioactive components.

Hippuric acid is gaining traction as a potential hallmark of aging and specifically for frailty and sarcopenia, with low plasma and urine hippuric acid concentrations associated with frailty and sarcopenia (55), and an inverse relationship evident between plasma hippuric acid concentration and the Frailty Index (56). This is highly pertinent since in the present study circulating hippuric acid concentrations, but not those of other phenolics were positively correlated with MVC, IKconcentric and IKeccentric values across study timepoints with typical Pearson r values of > 0.5. Hippuric acid is produced in the liver through conjugation of glycine with benzoic acid, a common metabolite produced by the microbial metabolism of flavonoids and chlorogenic acids such as caffeic acid derived from coffee (57). In view of its position as an end product of microbial metabolism of the majority of dietary polyphenols, it is perhaps unsurprising that plasma hippurate is indicative of diet quality, with high intakes of fruit and vegetables, as well as whole grains and coffee being associated with increased levels of hippurate (56, 58). Additionally, hippurate is indicative of microbial gene richness (59) and is suggested to be a metabolic marker of gut microbiome diversity (58). These data present the first empirical evidence of a relationship between muscle function and plasma hippurate in a young healthy population.

The present study is also the first to investigate the effects of tart cherry supplementation on the infiltration of immune cells within the exercised muscle following the damaging protocol. First, in line with previous literature investigating immunological changes in human skeletal muscle following intense exercise (13, 60), we found an increase in neutrophil infiltration of the exercised muscle following the damaging protocol, with values peaking at 24 h post. Tart cherry supplementation did not affect neutrophil infiltration into the exercised muscle. However, there was an effect of supplement on macrophage infiltration, with apparently higher macrophage infiltration at 48 h recovery in the low cherry condition. This is partly in agreement with previous rodent research where polyphenol supplementation with a dose achievable for human supplementation (equivalent to 140 mg.d^−1^ grape seed extract) led to an accelerated infiltration of macrophages in the injured tissue (61). More recent evidence suggests that amplification of early phase M1 macrophage infiltration enhances tissue healing (62). Tart cherry induced alterations in the time course and magnitude of the macrophage response to muscle damage may therefore contribute to enhanced functional recovery observed in previous studies, but this needs further investigation. Due to the selection of CD206 as cell marker we were not able to differentiate between M1 and M2 macrophages. Although, increasingly it is understood that rather than distinct subpopulations of M1 and M2 macrophages, there is a continuum of phenotypes with a shift toward a more predominant M2 anti-inflammatory population between day 1 and 3 of recovery (63).

Previous studies found a beneficial effect for tart cherry supplementation on functional recovery from intense physical exercise (17–19, 23, 28, 30, 31, 64), but that was not replicated in the present study. Two key factors may contribute to this discrepancy: firstly, differences in the supplementation strategy and secondly, a high drop-out rate and variability in the functional data. It is difficult to directly compare the polyphenol dose provided in the present study with previous studies due to the different analytical methods employed [gold standard UPLC-MS method in the present study vs. HPLC or Folin Ciocalteu methods in previous studies, see Sabou et al. (65) for review]. However, the dose of cherry concentrate provided in the high dose condition (60 ml) is on a par with previous studies (17, 19, 23) where favorable effects on functional recovery were observed. In the present study, there were two notable deviations from the supplementation protocol used in previous studies where functional recovery was enhanced. Firstly, the supplement was provided as a daily pre-diluted dose which needed to be refrigerated, rather than as a concentrate to be diluted by the participant. Secondly the daily dose was taken as a single morning bolus rather than being split into a morning and evening dose. Notwithstanding these supplementation protocol variations, the profound molecular changes induced within the skeletal muscle suggest that the dose was sufficient, and this conclusion is reinforced by the significant and dose dependent elevation in plasma phenolic metabolites.

To date, only Wangdi et al. (23) have published plasma phenolic metabolite concentrations in response to exercise-induced muscle damage and cherry supplementation (30 ml twice per day vs. 30 or 60 ml once per day in the present study). Most notably, the elevation in plasma hippuric acid was considerably smaller in the high dose condition in the present study after a 7 d pre-load (~2-fold elevation) than in the Wangdi et al. (23) study (>3-fold elevation). *Post hoc* comparisons indicated that the significant difference in plasma hippuric acid existed only between high and low dose conditions and not vs. placebo, prior to the muscle damaging protocol. It is plausible that this attenuated plasma hippuric acid response to cherry supplementation in the present study contributed to the absence of functional effects, since plasma hippuric acid is correlated with preservation of muscle strength and lower risk of frailty in the elderly (55, 56). There was also a strong positive association between plasma hippuric acid and functional data in the present study. Participants randomized to the placebo condition appear to have high background levels of circulating hippuric acid and this may have contributed variability to their functional data. Plasma hippuric acid is suggested to be indicative of gut microbial alpha diversity (59), so will vary across individuals. Additionally, the interaction between tart cherry polyphenols and gut microbiome may have been compromised by the large sugar load accompanying the single 60 ml dose resulting in a lower hippuric acid response and potentially attenuating the ergogenic effects (66). Lastly, the dropout rates in our study (30.6%) were substantially higher compared to our previous study which deployed a similar protocol [Wangdi et al. (23), 16.7%]. A potential contributor to this high drop-out rate was the substantially larger size of the trial (34 participants) compared to previous studies where only 10–12 participants completed this protocol (18, 23). This challenge was further accentuated by the difficulties of conducting human trials during the COVID-19 pandemic and retaining participants in the period following a return to human participant testing (67). These factors also likely played a key role in the high variability seen in the functional data and may have contributed to the clear statistical need to eliminate six participants from the functional data analyses. Indeed, in the Wangdi et al. (23) study in which an identical exercise protocol was used, the variability in the reduction in MVC immediately post damaging exercise was much smaller (*n* = 10; coefficient of variation: 12.8%; range 46–88%) than in the present study (*n* = 34: coefficient of variation: 22.7%; range 34–96%; with 6 participants excluded: *n* = 28: SD/mean: 13.6%; range 38–91%).

## 5 Limitations of the study

The current study significantly advances our understanding of the impact of tart cherry supplementation and exercise-induced muscle damage on the skeletal muscle proteome. However, there are some limitations that should be summarized; many of these have already been alluded to in the previous section. First, we assessed recovery from EIMD for up to 48 h. However, remodeling of skeletal muscle after such damage can last for up to 96 h, with “anti-inflammatory” macrophages thought to predominate beyond 48 h.

Second, we measured 6 phenolic metabolites in the plasma, chosen based on previous literature. Although our analyses yielded important insights regarding the relationships between these metabolites and functional/proteomic outcomes, a more comprehensive characterization of the plasma phenolic signature may have added further depth to these analyses.

Lastly, the high participant drop-out rate, and variability in the muscle performance data compromised our ability to identify functional changes. We suggest that these factors were driven by the post-COVID research environment.

## 6 Conclusion

Tart cherry supplementation led to differential changes in skeletal muscle expression of structural and contractile proteins in response to eccentric exercise. Increased expression of these proteins after a 7 d tart cherry pre-load may be protective in skeletal muscle subjected to subsequent damaging exercise, although recovery of function was not enhanced in the present study. There is intense involvement of extracellular matrix proteins in the muscle damage response, and tart cherry increased expression of structural proteins 48 h post-exercise. Notably, plasma hippuric acid was positively associated with knee extensor strength and proteins in mitochondrial and translational pathways. implicating hippurate as a mediators or biomarker of tart cherry bioactivity *in vivo*. Importantly, this is the first demonstration of plasma hippuric acid association with skeletal muscle strength in a young healthy population. This has previously been demonstrated in frail older populations. These data provide important insights into the mediators of tart cherry effects in skeletal muscle, and strong potential for therapeutic and training adaptation applications.

## Data Availability

The raw data analyzed in this study can be found here: https://osf.io/2ad76?view_only=af5a80b4b85a4aadb2c119593df99355.
