# Supplementary material for: Tart cherry supplementation causes differential regulation of skeletal muscle proteome after eccentric exercise
Source: Front Nutr. 2026 May 26;13:1801399. doi: 10.3389/fnut.2026.1801399 (PMC13247740; doi:10.3389/fnut.2026.1801399)
Supplement: Supplementary file 1 [file Table_1.docx]

Supplementary Material

Supplementary Material A

Table S1. Tart cherry concentrate analysis for phenolic composition conducted at the Quadram Institute utilising HPLC (Holland et al, 2017) and UPLC- MS (Wojdylo et al, 2014) methods.

| Phenolic compound | Average Content (mg/L) |
| --- | --- |
| Procyanidin  Chlorogenic acid  Epicatechin  Neochlorogenic acid  Rutin  p-coumaric acid  Catechin  Caffeic acid  Vanillic acid  Ferulic acid | 4.9  857.5  437.0  390.8  181.9  104.4  59.4  42.6  35.5  11.1 |

Table S2. Stability of tart cherry concentrate anthocyanin content (mg/L) of the assessed every 3 months through the study duration using UPLC-MS with UV-VIS detection.

| Analysis Date | Cyanidin 3-glucosylrutinoside | Cyanidin 3-O-rutinoside | Peonidin 3-O-rutinoside | Total |
| --- | --- | --- | --- | --- |
| Jan 2022  April 2022  June 2022  Aug 2022  Dec 2022 | 434.9  444.4  448.9  414.9  385.4 | 106.4  102.9  103.9  100.3  97.0 | 10.2  10.2  10.5  10.5  9,7 | 551.6  557.7  565.4  525.6  492.0 |

Table S3. Plasma phenolic metabolite assay characteristics

|  | **Protocatechuic acid**  **nmol/L** | **4-hydroxybenzoic acid**  **nmol/L** | **hippuric acid**  **µmol/L** | **vanillic acid**  **nmol/L** | **ferulic acid**  **nmol/L** | **isoferulic acid**  **nmol/L** |
| --- | --- | --- | --- | --- | --- | --- |
| Linearity | 0.5-486 | 1.0-749 | 0.01-800 | 0.5-336.7 | 0.5-321.1 | 0.5-337.7 |
| Typical r^2^ | >0.98 | >0.98 | >0.98 | >0.98 | >0.98 | >0.98 |
| Intra assay imprecision conc. mean (%CV), n=6 | 39.4 (2.6)  79.4 (5.8)  430.4 (2.2) | 57.3 (3.5)  261.6 (2.5)  622.9 (2.0) | 6.1 (0.3)  34.7 (0.9)  499.2 (2.4) | 36.9 (9.5)  43.7 (5.9)  250.9 (7.6) | 3.3 (6.6)  9.4 (3.7)  143.9 (2.1) | 2.1 (4.0)  106.2 (4.2)  218.6 (4.4) |
| Inter assay imprecision  conc. mean (%CV), n=6 | 20.0 (10.6)  135.1 (9.0)  424.7 (7.0) | 32.9 (6.0)  393.6 (6.2)  622.8 (7.6) | 32.9 (6.0)  393.6 (6.2)  522.8 (7.6) | 35.7 (5.4)  221.6 (6.8)  359.4 (9.2) | 4.5 (6.1)  132.2 (6.1)  294 (7.0) | 3.7 (9.9)  133.6 (6.5)  277 (7.5) |
| Lower Limits of quantification (LLoQ) | 1.0 | 1.0 | 0.05 | 1.0 | 1.0 | 1.0 |
| Spiked recovery Mean% (±SD)* | 98.5% (±2) | 96.8% (±3) | 103% (±2) | 105% (±2) | 99% (±2) | 102% (±3) |
| * Base serum used for spiking contained 100 µmol.L^-1^ of hippuric acid and no other endogenous phenolic metabolites. Each spiked sample was tested six times. | | | | | | |

### Supplementary Material B.

Table S3. Muscle function raw data for MVC1s, MVC, IK^ECC^ and IK^Con^ in the three conditions (PLA, LTC and HTC) at the following timepoints: pre-damaging protocol, immediately after, 24h and 48h after the damaging protocol. Values are presented as means ± SD.

| **Measure** | **Supplement** | **Pre-exercise** | **Post-exercise** | **24h** | **48h** | **Condition Effect (P)** | **Time Effect (P)** |
| --- | --- | --- | --- | --- | --- | --- | --- |
| MVC1s (N) | PLA | 272.8±53.8 | 173.5±58.4 | 192.6±59.6 | 219.4±69.0 | Non-significant (p=0.067) | Significant (p<0.001) |
|  | LTC | 272.5±60.0 | 176.7±44.9 | 189.1±45.3 | 203.6±55.8 |  |  |
|  | HTC | 272.6±61.1 | 182.1±51.1 | 189.1±58.3 | 205.8±54.8 |  |  |
| MVC (N) | PLA | 279.7±53.5 | 182.5±57.1 | 202.4±63.6 | 227.0±70.1 | Non-significant (p=0.085) | Significant (p<0.001) |
|  | LTC | 282.3±57.8 | 186.3±46.3 | 195.5±45.6 | 211.4±56.4 |  |  |
|  | HTC | 281.3±62.6 | 193.7±51.1 | 196.7±59.6 | 212.0±56.2 |  |  |
| IK^Ecc^ (N) | PLA | 255.4±56.3 | 197.1±64.8 | 197.9±68.8 | 219.0±80.3 | Non-significant (p=0.231) | Non-significant (p=0.086) |
|  | LTC | 257.8±67.6 | 198.4±44.7 | 192.6±53.7 | 201.3±64.7 |  |  |
|  | HTC | 263.3±54.4 | 190.8±46.1 | 201.6±54.7 | 209.0±54.1 |  |  |
| IK^Con^ (N) | PLA | 179.5±34 | 138.0±45.5 | 138.5±45.4 | 150.5±44.2 | Non-significant (p=0.138) | Non-significant (p=0.056) |
|  | LTC | 189.9±43.8 | 140.5±34.3 | 139.2±23.5 | 145.3±30.2 |  |  |
|  | HTC | 194.9±37.3 | 156.6±32.8 | 148.8±31.4 | 155.2±31.5 |  |  |

### Supplementary Material C.

Table S4. Table indicating the Pearson r and P values for the correlations between Hippuric Acid and the muscle function tests carried out prior, immediately post, 24h and 48h post the muscle-damaging protocol.

|  | **Pearson r** | | | | **P value** | | | |
| --- | --- | --- | --- | --- | --- | --- | --- | --- |
|  | **Hippuric Acid** | | | | | | | |
|  | **Baseline** | **PostEx** | **24h** | **48h** | **Baseline** | **PostEx** | **24h** | **48h** |
| **MVC1s Baseline** | 0.41 | 0.49 | 0.48 | 0.58 | 0.030 | 0.008 | 0.010 | 0.001 |
| **MVC1s PostEx** | 0.61 | 0.64 | 0.53 | 0.63 | 0.001 | 0.000 | 0.003 | 0.000 |
| **MVC1s 24h** | 0.59 | 0.61 | 0.58 | 0.64 | 0.001 | 0.001 | 0.001 | 0.000 |
| **MVC1s 48h** | 0.50 | 0.53 | 0.38 | 0.67 | 0.006 | 0.004 | 0.046 | 0.000 |
| **MVC Baseline** | 0.41 | 0.49 | 0.42 | 0.55 | 0.028 | 0.008 | 0.024 | 0.002 |
| **MVC PostEx** | 0.60 | 0.62 | 0.51 | 0.67 | 0.001 | 0.000 | 0.005 | 0.000 |
| **MVC 24h** | 0.62 | 0.62 | 0.57 | 0.66 | 0.000 | 0.000 | 0.002 | 0.000 |
| **MVC 48h** | 0.49 | 0.51 | 0.36 | 0.68 | 0.008 | 0.005 | 0.064 | 0.000 |
| **IKEccentric Baseline** | 0.31 | 0.53 | 0.56 | 0.16 | 0.106 | 0.003 | 0.002 | 0.402 |
| **IKEccentric PostEx** | 0.44 | 0.46 | 0.56 | 0.58 | 0.020 | 0.013 | 0.002 | 0.001 |
| **IKEccentric 24h** | 0.53 | 0.52 | 0.59 | 0.63 | 0.004 | 0.004 | 0.001 | 0.000 |
| **IKEccentric 48h** | 0.30 | 0.31 | 0.38 | 0.60 | 0.119 | 0.108 | 0.047 | 0.001 |
| **IKConcentric Baseline** | 0.24 | 0.35 | 0.55 | 0.59 | 0.214 | 0.066 | 0.002 | 0.001 |
| **IKConcentric PostEx** | 0.53 | 0.48 | 0.68 | 0.61 | 0.004 | 0.009 | 0.000 | 0.001 |
| **IKConcentric 24h** | 0.57 | 0.55 | 0.64 | 0.63 | 0.002 | 0.002 | 0.000 | 0.000 |
| **IKConcentric 48h** | 0.42 | 0.41 | 0.46 | 0.70 | 0.028 | 0.030 | 0.014 | 0.000 |
